# Expression of GM content in mass fraction from digital PCR data

**DOI:** 10.1016/j.foodcont.2021.108626

**Published:** 2022-03

**Authors:** Philippe Corbisier, Gerhard Buttinger, Cristian Savini, Maria Grazia Sacco, Francesco Gatto, Hendrik Emons

**Affiliations:** a European Commission, Joint Research Centre JRC, Retieseweg 111, 2440, Geel, Belgium; b European Commission, Joint Research Centre JRC, Via Enrico Fermi 2749, 21027, Ispra, VA, Italy

**Keywords:** Genetically modified organism, Digital PCR, Certified reference material, Unit of measurement, Quantification, Conversion factor

## Abstract

Nowadays the quantification of the content of genetically modified (GM) constituents in food or feed products is performed by using either quantitative real-time PCR (qPCR) or digital PCR (dPCR). The latter is increasingly used. Therefore, experimental protocols for the quantification of 52 GM events authorised in the EU have been converted into a digital format and minimum performance characteristics for dPCR methods are detailed. Because of the need to harmonise the transformation of PCR results between two different measurement scales, 50 conversion factors for Certified Reference Materials (CF_CRM_) have been experimentally determined by three and sometimes four independent expert laboratories. The uncertainty of each CF_CRM_ has been estimated to express dPCR results in mass fraction with a consistent uncertainty contribution. In 38 out of 58 cases, the validated qPCR methods (for 52 event-specific and 6 taxon-specific measurements) could easily be transferred into dPCR methods by using the same oligo sequences, final oligo concentration or annealing temperatures for the dPCR procedure. Laboratories have nevertheless used different strategies to improve the resolution or to reduce the so-called rain in their dPCR outcome. Those modifications were needed for PCR procedures that could not be converted without changes into a digital format. Therefore, exclusion/quality criteria such as the maximum rate of partitions with intermediate fluorescence “rain”, the minimum resolution and repeatability are suggested for dPCR methods. The CF_CRM_ determined in this study were generally in agreement with the declared zygosity of the GM parental donor for hemizygous maize events. In a limited number of GM events the CF_CRM_ values were significantly different when measured with different maize-specific (*ZmAdh1* or *hmgA*) genes.

## Highlights

• Digital PCR protocols for the quantification of 52 GM events
• Minimum performance criteria for digital PCR results
• Conversion factors to express GM content determined by digital PCR in mass fraction
• Harmonised measurement system for official GMO controls in the EU

## 1 Introduction

In the European Union procedures (EC Regulation, 2003) and detailed implementation rules (EC Regulation, 2004) for the authorisation and monitoring of genetically modified (GM) food and feed, including legal provisions for the labelling of such products, are applied. The corresponding requirements on applications for marketing authorisation include the provision of methods for the detection, identification and quantification of the GM product as well as information where the related reference material can be obtained (EU Regulation, 2013).

Both the reference material and the specified detection method are part of a reference system, which provides unambiguous measurement results (Trapmann, Corbisier, & Schimmel & Emons, 2010). It is scientifically straightforward that measurement results obtained by a quantitative real-time polymerase chain reaction (qPCR) method calibrated with a particular certified reference material (CRM) should be expressed in the measurement unit in which the property value of the CRM is certified. For official control purposes in the EU Regulation (EC) No 641/2004 specifies that the GMO content of the CRM shall be given in mass fraction (EC Regulation, 2004). Moreover, Regulation (EU) No 619/2011 explains that the measurement result for the analysis of GM feed for which an authorisation procedure is pending or its authorisation has expired has to be presented in mass fraction (EU Regulation, 2011).

The European Reference Laboratory for GM Food and Feed (EURL GMFF), assisted by the European Network of GMO Laboratories (ENGL), has been mandated within the authorisation procedure to validate the detection methods and to define and verify performance characteristics of the methods submitted by the applicants. All the detection methods published by the EURL GMFF within the frame of the Regulation (EC) No 1829/2003 are based on qPCR which has been recognised for many years as the best available quantification technique (EURL GMFF, 2021).

However, digital polymerase chain reaction (dPCR) (Vogelstein & Kinzler, 1999) has become another mainstream technology over the last 10 years and has complemented existing DNA analysis methods based on qPCR for the quantification of nucleic acid targets. Different dPCR instrumentation has been designed to offer an alternative to qPCR and an increasing number of laboratories have converted their validated qPCR procedures into a dPCR format.

The fundamental difference between qPCR and dPCR consists in how the amounts of target sequences are measured and evaluated. In qPCR, the reaction product is monitored during the amplification process and the quantification is based on a fluorescence signal in the exponential part of the signal intensity-concentration relation. In contrast, dPCR collects fluorescence signals via an end-point detection of the amplification process and uses the number of subsamples showing fluorescence (positive partitions) in comparison to the total number of measured subsamples (partitions) to calculate the target DNA concentration via a statistical model. Unlike qPCR, dPCR does not rely on DNA-based calibration curves for quantification and avoids the pitfalls associated with the variations in reaction efficiencies (Quan, Sauzade, & Brouzes, 2018). Consequently, dPCR methods were also shown to be less sensitive to PCR inhibitors compared to qPCR (Iwobi, Gerdes, Busch, & Pecoraro, 2016) which is considered as an advantage when using sub-optimal DNA extraction methods on difficult matrices.

Some pioneering studies have investigated the experimental parameters that affect the accuracy of measurement results obtained by dPCR (Bhat et al., 2009, 2010; Corbisier et al., 2015; Emslie et al., 2019; Pinheiro et al., 2012) and the related sources of uncertainties (Deprez et al., 2016). The availability of specific dPCR guidelines to improve both the reporting and reviewing of scientific papers (ThedMIQE Group & Huggett, 2020) as well as an international standard providing the requirements for the evaluation of the performance of quantification methods for nucleic acid targets sequences by qPCR and dPCR (ISO 20395, 2019) indicate a certain degree of maturity for this methodology.

In the context of quantifying genetically modified organisms (GMO) in food and feed products a proof of concept showed that the copy number concentration of GM maize MON810 and the maize taxon-specific targets *hmg* present in a DNA extract from seed powders, certified for their GM mass fraction and for the DNA copy number ratio transgene/taxon-specific, could be measured by dPCR (Corbisier, Bhat, Partis, Xie, & Emslie, 2010). It was also demonstrated that the ratio of these copy numbers determined by dPCR was identical to the ratio measured by real-time quantitative PCR (qPCR) using a plasmid DNA calibrant. Moreover, the parental origin of the MON810 trait could be verified by dPCR. The equivalence of measurement results obtained by dPCR and qPCR using an appropriate plasmidic calibrant has also been demonstrated by other studies (Burns et al., 2006; Caprioara-Buda et al., 2012) including interlaboratory studies on different plant matrices (Corbisier et al., 2012; Mester et al., 2020). The applicability of dPCR for the assessment of detection limits in GMO analysis has been reviewed (Burns, Burrell, & Foy, 2010) and dPCR methods were successfully applied on food and feed samples in simplex (Morisset, Štebih, Milavec, Gruden, & Žel, 2013), duplex (Félix-Urquídez et al., 2016) and multiplex formats (Dobnik, Spilsberg, Bogožalec, Holst-Jensen, & Žel, 2015; Dobnik, Štebih, Blejec, Morisset, & Žel, 2016).

However, the quantification of GMO by a dPCR method instead of a qPCR method has two major consequences.

At first, dPCR data provide the number of measurable DNA fragments (copies) and the GMO content of a sample could be directly expressed as a DNA copy number ratio of a GM event-specific target and a taxon (or species)-specific target. However, such a measurement result has to be converted into a mass fraction for the enforcement of EU legislation. To realise this conversion, the DNA copy number ratio embedded in a particular CRM, which is certified for its GM mass fraction, could be determined with sufficiently precise dPCR measurements. On this basis a CRM-specific conversion factor (CF_CRM_) can be established as the ratio of the number of DNA copies of the transgenic sequence divided by the number of DNA copies of the taxon-specific sequence present in the CRM made available for the authorisation process (Corbisier et al., 2017). Thereby, a reference measurement system is available for GM quantification composed of three elements (components): one reference qPCR method per GM event (validated according to ISO/IEC 17025), one CRM per GM event (produced according to ISO 17034) and one conversion factor per CRM (CF_CRM_) to transform dPCR measurement data into a mass fraction result (Corbisier & Emons, 2019).

Secondly, each qPCR method that was validated and published by the EURL GMFF as reference method needs to be converted into a dPCR method. In principle the same DNA targets can be amplified by PCR using the same primers and probe sequences. But adjustments with respect to the reagents, optimised oligo concentrations, prior digestion of the DNA, and annealing temperature may be required for the optimised differentiation of positive and negative partitions/droplets in dPCR (Pecoraro et al., 2019).

In the work reported here, dPCR experimental protocols for the quantification of 52 GM events authorised in the EU have been optimised, the minimum performance characteristics for dPCR results were specified and 50 CF_CRM_ have been experimentally determined by at least three and up to four independent expert laboratories and their uncertainties were estimated.

## 2 Materials and methods

### 2.1 Materials

Certified reference materials (CRMs) listed in the Annexes of Commission Decisions authorising (or renewing an authorisation of) the placing on the market of products containing, consisting of, or produced from genetically modified plants were obtained from the Joint Research Centre (JRC, Geel, Belgium) or purchased from the American Oil Chemists’ Society (AOCS, Urbana, IL, USA), see Table 1. When available, CRMs with the highest GM content were systematically purchased, namely for maize: ERM®-BF415f (NK603), ERM®-BF423d (MIR604), ERM®-BF433d (DAS-4Ø278-9), ERM®-BF420c (3272), ERM®-BF438e (VCO01981), ERM®-BF416d (MON863), ERM®-BF412bk (Bt11), ERM®-BF424d (DAS59122), ERM®-BF418d (1507), ERM®-BF411f (Bt176) and 0906-E (MON89034), 1208-A2 (MIR162), 0709-A (MON87460), 0512-A (MON87427), 0411-D (5307), 0407-B (GA21), 0306-H9 (T25), 0406-D (MON88017); for cotton: ERM®-BF422d containing the stacked events (281-24-236) and (3006-210-23), ERM®-BF429c (T304-40), ERM®-BF428c (BCS-GHØØ5-8) and 0113-A (MON 88701), 0306-E2 (LLCotton25), 0804-B (MON1445), 0804-D (MON15985), 0804-C (MON 531), 1108-A5 (GHB614), 0906-D (MON88913); for soybean: ERM®-BF432d (DAS-68416-4), ERM®-BF437e (DAS-81419-2), ERM®-BF425d (356034), ERM®-BF436e (DAS-44406-6), ERM®-BF410ep (40-3-2), ERM®-BF426d (305423) and 0906-B (MON89788), 0809-A (MON87701), 0707-C6 (A5547-127), 0210-A (MON87705), 0311-A (MON 87708), 0809-B (MON 87769), 0911-D (BPS-CV127-9), 0610-A4 (FG 72), 0707-B10 (A2704-12); for rapeseed: ERM®-BF434e (73496), 0306-F6 (MS8), 0306-G5 (RF3), 0304-B2 (GT73 or RT73), 0208-A5 (T45), 1011-A (MON-883Ø2-9) for rice: 0306-I8 (LLRice62) and for sugar beet: ERM®-BF419b (KM- ØØØ71-4). All CRMs from the JRC were sent centrally to the contracted laboratories, whereas each laboratory purchased the CRMs from AOCS individually. The CRMs consisting of a DNA solution from GM leaves were all purchased from AOCS and kept at 4 °C until use.

**Table 1:** Coding, description, type of material, certified value and associated uncertainty of the CRMs tested in this study (provider information).

CRM Code	Description	Type of material	Certified value	Uncertainty
**0306-H9**	T25 Maize	gDNA from leaves	>999.99 ng/μg	0 ng/μg
**0306-I8**	LLRice62 rice	gDNA from leaves	pure	n/a
**0306-E2**	LLCotton25 cotton	gDNA from leaves	pure	n/a
**1108-A5**	GHB614 cotton	gDNA from leaves	>999.99 ng/μg	0 ng/μg
**0707-B10**	A2704-12 soybean	gDNA from leaves	pure	n/a
**0707-C6**	A5547-127 soybean	gDNA from leaves	pure	n/a
**0610-A4**	FG72 soy	gDNA from leaves	pure	n/a
**0306-F6**	Ms8 canola	gDNA from leaves	pure	n/a
**0306-G5**	Rf3 canola	gDNA from leaves	pure	n/a
**0208-A5**	T45 canola	gDNA from leaves	pure	n/a
**0407-B**	GA21 maize	seed powder	>991.5 g/kg	with 95% confidence
**0406-D**	MON88017 maize	seed powder	pure	n/a
**0906-E**	MON89034 maize	seed powder	1000 g/kg	>985.1 g/kgwith 95% confidence
**1208-A2**	MIR162 maize	seed powder	>991.5 g/kg	with 95% confidence
**0709-A**	MON87460 maize	seed powder	pure	n/a
**0512-A**	MON87427 maize	seed powder	1000 g/kg	>994.8 g/kgwith 95% confidence
**0411-D**	5307 maize	seed powder	>982.2 g/kg	with 95% confidence
**0113-A**	MON88701 cotton	seed powder	>984.5 g/kg	NA
**0804-B**	MON1445 cotton	seed powder	pure	n/a
**0804-D**	MON15985-7 cotton	seed powder	pure	n/a
**0804-C**	MON531 cotton	seed powder	pure	n/a
**0906-D**	MON88913 cotton	seed powder	pure	n/a
**0906-B**	MON89788 soybean	seed powder	pure	n/a
**0809-A**	MON87701 soybean	seed powder	1000 g/kg	>991.5 g/kgwith 95% confidence
**0210-A**	MON87705 soybean	seed powder	pure	n/a
**0311-A**	MON87708 soybean	seed powder	997.2 g/kg	>986.6 ng/gwith 95% confidence
**0809-B**	MON87769 soybean	seed powder	pure	n/a
**0911-C/D**	CV127 soybean	seed powder	1000 g/kg	>963.2 g/kgwith 95% confidence
**0304-B2**	GT73/RT73 canola	seed powder	pure	n/a
**1011-A**	MON88302 canola	seed powder	pure	n/a
**ERM®-BF412bk**	Bt-11 maize (level 5 – nominal 5% GMO)	seed powder	>970 g/kg	with 95% confidence
**ERM®-BF424d**	59122 maize (level 3 – nominal 10% GMO)	seed powder	98.7 g/kg	- 5.8 g/kg
				+5.9 g/kg
**ERM®-BF418d**	1507 maize (level 3 – nominal 10% GMO)	seed powder	98.6 g/kg	- 1.7 g/kg
				+2.0 g/kg
**ERM®-BF415f**	NK603 maize (level 5 – nominal 5% GMO)	seed powder	49.1 g/kg	1.3 g/kg
**ERM®-BF423d**	MIR604 maize (level 3 – nominal 10% GMO)	seed powder	98.5 g/kg	- 2.6 g/kg
				+2.9 g/kg
**ERM®-BF433d**	DAS-40278-9 maize (nominal 10% GMO)	seed powder	100 g/kg	8 g/kg
**ERM®-BF420c**	3272 maize (level 2 – nominal 10% GMO)	seed powder	98 g/kg	8 g/kg
**ERM®-BF438e**	Genetically modified VCO- Ø1981-5 maize (nominal 10% GMO)	seed powder	100 g/kg	5 g/kg
**ERM®-BF411f**	Bt-176 maize (level 5 – nominal 5% GMO)	seed powder	50.0 g/kg	1.8 g/kg
**ERM®-BF416d**	MON 863 maize (level 3 – nominal 10% GMO)	seed powder	98.5 g/kg	- 2.2 g/kg+2.5 g/kg
**ERM®-BF422d**	281-24-236 × 3006-210-23 cotton seed (level 3 – nominal 10% GMO)	seed powder	100 g/kg	16 g/kg
**ERM®-BF429c**	T304-40 cotton (level 2 – nominal 10 5 GMO)	seed powder	100 g/kg	11 g/kg
**ERM®-BF428c**	Cotton GHB119 (level 2 – nominal 10% GMO)	seed powder	100 g/kg	11 g/kg
**ERM®-BF432d**	Soya DAS-68416-4 (level 3 – nominal 10% GMO)	seed powder	100 g/kg	13 g/kg
**ERM®-BF436e**	DAS-44406-6 soya (nominal 10% GMO)	seed powder	100 g/kg	9 g/kg
**ERM®-BF437e**	DAS-81419-2 soya (nominal 10% GMO)	seed powder	100 g/kg	9 g/kg
**ERM®-BF410ep**	GTS 40-3-2 soya bean (level 3 – nominal 10% GMO)	seed powder	100 g/kg	5 g/kg
**ERM®-BF425d**	Soya 356043 (level 3 – nominal 10 5 GMO)	seed powder	100 g/kg	9 g/kg
**ERM®-BF426d**	Soya 305423 (level 3 – nominal 10% GMO)	seed powder	100 g/kg	7 g/kg
**ERM®-BF434e**	73496 rapeseed (nominal 10 5)	seed powder	100 g/kg	12 g/kg
**ERM®-BF419b**	H7-1 sugar beet (level 1 – nominal 100% GMO)	seed powder	1000 g/kg	0 g/kg

From the 51 CRMs, 10 CRMs consisting of heterogeneous mixtures of coarse and fine particles were not considered as sufficiently homogeneous by Laboratory 1. Visible chunks and coarse pieces of seeds were found in the maize CRMs coded 0406-D, 709-A, 0906-E, 1208-A, 0512-A and 0411-D; in the cotton CRMs coded 0113-A and 0906-D; and in the soybean CRMs coded 0311-A and 0911-D. Those CRMs were therefore first re-milled using a Retsch ball mill (Haan, DE) in dry ice during 15 s at a frequency of 30 Hz and sieved on a stainless steel sieve with a mesh size of 710 μm. The resulting ground powder was sieved again through a stainless steel sieve with 710 μm mesh size. The process was repeated until all the material passed through the 710 μm sieve. The milling was considered as particularly important to guarantee a homogeneous ratio of GM and non-GM targets for the plant tissues of hemizygous crops.

### 2.2 Study design

**Fig. 1:**
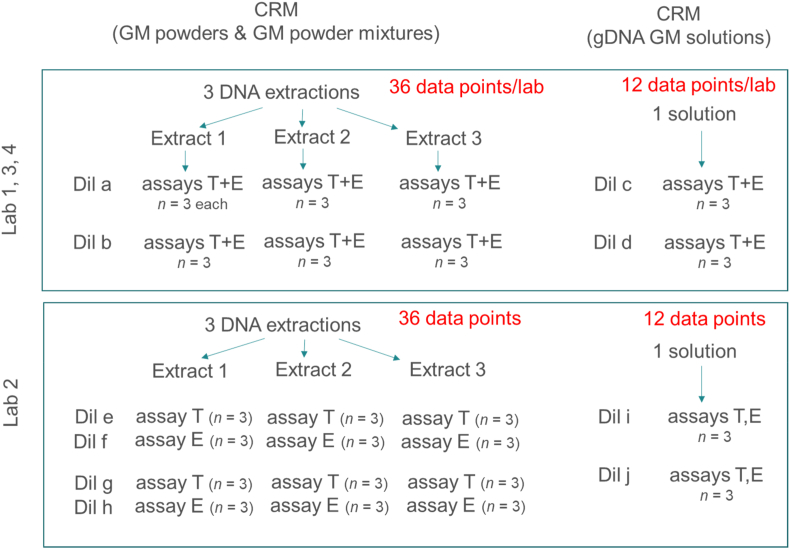
Experimental design followed in this study. The four laboratories (L1, L2, L3 and L4) extracted DNA from the powder CRMs following their optimised extraction method. Three independent extracts were analysed by qPCR with the Transgenic (T) and taxon-specific assays (E) in triplicated wells. The Lab 2 tested the three DNA extracts at 4 dilution levels (Dil e, Dil f; Dil g and Dil h) using independent dilutions for the transgenic and taxon-specific assays, whereas L1, L3 and L4 tested the transgenic and taxon-specific assays at two dilution levels (Dil a and Dil b). A maximum of 36 data points per laboratory per assay could be obtained for the powder CRMs, whereas 12 data points per laboratory per assay were expected for the analysis of the genomic DNA solution CRMs.

Three laboratories had been invited to measure the DNA copy number ratio of 51 CRMs covering 52 unique GM events and their corresponding taxon-specific target. In a second stage, a fourth laboratory was asked to re-measure 18 CRMs for a confirmation of the results obtained. The CRMs consisted of powder materials or genomic DNA solutions as shown in the schematic study design (Fig. 1). The laboratories were selected on the basis of their demonstrated ability to perform dPCR measurements with the necessary precision. Despite the low number of participating laboratories, the study was designed in such a way that it should provide an acceptable low uncertainty associated to the determination of the CF. The four laboratories had to perform dPCR using instrumentation from Bio-Rad for the generation and reading of the droplets. The CRMs consisting of genomic DNA (gDNA) solutions had to be measured in two dilutions each for the transgene and the taxon-specific targets. The dilutions were designed for being able to detect potential analytical issues such as inhibition effects or inconsistencies between dilutions. Each dilution had to be measured in triplicate resulting in 6 measurements each for the transgene and the taxon-specific targets. gDNA was extracted from each powder CRM in three independent extractions. Each laboratory could use the most appropriate extraction method in dependence of the nature of the matrix analysed (see below). If deemed necessary, laboratories had the possibility to increase the sample intake or to firstly grind samples considered as inhomogeneous. However, Laboratory L1 was the only laboratory that ground 10 CRMs considered as not sufficiently homogeneous. Each of the 3 extracts had to be analysed at two dilution levels providing 6 measurement data for the transgenic targets and 6 measurement data for the taxon-specific target. Per powder CRM, a total of 18 data points were generated for the transgenic target and 18 data points for the taxon-specific target.

### 2.3 DNA extraction methods

Laboratory 1 (L1): The gDNA was extracted from 200 mg powders using a CTAB method including a Genomic-tip 20/G clean-up (Qiagen®, Hilden DE). The CTAB method was optimised for the different taxa. The DNA extraction performance was assessed via agarose gel electrophoresis and UV spectrophotometry (Nanodrop, Thermo Scientific™).

Laboratory 2 (L2): Three DNA extractions protocols were used depending on the taxa and the provider of the CRMs. For maize samples, DNA was extracted by a CTAB method from either 200 mg of the material (ERM®-BF412f, ERM®-BF424d, ERM®-BF418d, ERM®-BF415f, ERM®-BF423d, ERM®-BF433d, ERM®-BF420c, ERM®-BF438e, ERM®-BF411f, ERM®-BF416d) or by a NucleoSpin Food kit (Machery-Nagel, MN-740945.50) from 1 g of the material (0406-D, 0906-E, 1208-A, 0709-A, 0512-A, 0411-D, 0407-B). For soybean, oilseed rape and sugar beet a CTAB method was used, and DNA was extracted either from 200 mg (ERM®-BF432d, ERM®-BF436e, ERM®-BF437e, ERM®-BF410ep, ERM®-BF425d, ERM®-BF426d, ERM®-BF434e, ERM®-BF419b) or 1 g (0906-B, 0809-A, 0210-A, 0311-A, 0809-B, 0911-D, 0304-B2, 1011-A) of sample. A CTAB/genomic tip 20 was used for extraction of DNA from cotton samples of 200 mg (ERM®-BF428c, ERM®-BF422d, ERM®-BF429c) or 1 g (0113-A, 0804-B, 0804-D, 0804-C, 0906-D). The DNA extractions were performed under repeatability conditions.

Laboratory 3 (L3): DNA extractions were performed using a Maxwell®RSC Pure Food GMO (Promega) and Authentication kit following the manufacturer's instructions (Cat AS1600, Promega) with a sample intake of 100 mg for powder materials. The extracted DNA was suspended in 100 μL elution buffer. Briefly, 100 ± 10 mg of each material was weighted. The samples were extracted in batches of 12. Each batch consisted of four different CRMs in triplicate separated by blank extracts. Where possible, the four samples of each batch were belonging to four different taxa. An RNase treatment step was performed and samples were eluted in 100 μL of elution buffer.

Laboratory 4 (L4): DNA extractions were performed using a CTAB-based extraction procedure, a Nucleospin Food kit (Macherey-Nagel, Düren, DE) or the Foodproof GMO Sample Preparation kit III (Biotecon Diagnostics GmbH, Potsdam, DE) following manufacturer's instructions from soybean, maize and cotton/rapeseed powder CRMs, respectively. The sample intake was 200 mg for the three extraction methods and the DNA was eluted in 100 μL TE buffer or elution buffer.

### 2.4 Total DNA quantification

The genomic double stranded DNA (dsDNA) concentration extracted from the powder CRMs was estimated by fluorometry with the Picogreen® dsDNA quantitation kit (Molecular Probes-Invitrogen, Carlsbad, CA, USA) by L1 and L4 or with the QuantiFluor dsDNA System (Promega) by L3 following manufacturer's instructions. L2 estimated the DNA content in the extract by using a Nanodrop ND-1000 spectrophotometer (NanoDrop Technologies, Wilmington, DE). The genomic DNA solutions were diluted in Nuclease free water or 10 mM Tris-HCl (pH 8.0) + 0.1 mM EDTA when needed and kept at 4 °C.

### 2.5 Enzymatic restriction

All DNA solutions (except for NK603) extracted from maize powder CRMs have been restricted by *Eco*RI during 60 min at 37 °C by laboratory L1 to reduce the number of droplets which produce a signal between the clusters of signals from negative and positive droplets, also called “rain”, observed with the *hmg* assay. L1 verified that none of the targeted amplicons contained an *Eco*RI restriction site that would kill the targets amplifiability. Genomic DNA solutions from the rapeseed CRMs 0208-A5 (event T45), 0306-F6 (event Ms8) and 0306-G5 (event Rf3) were also restricted by EcoRV during 60 min at 37 °C, whereas DNA extracted from MON531 cotton was restricted by *Mse*I. The specific restriction enzymes were chosen after having verified *in silico* that no restriction sites were present in the respective amplicons. The restriction enzymes are later inactivated during the first activation step of the Taq polymerase.

### 2.6 Oligonucleotides

The sequences of the primers and probes (Supplementary material, Tables S1.3-7) were chosen from the European Reference Method Database (2021GMOMETHODS). The oligonucleotides were purchased by each laboratory from their preferred providers. The probes were labelled with the fluorophore as described in each EURL GMFF method validation report unless mentioned differently in Tables S1–5 and marked in red. The taxon-specific reference targets selected (Table S1.3) are those recommended by the EURL GMFF for qPCR methods, namely the high mobility group protein A (*hmgA)* gene for maize, *lectin*1 gene for soya, *FatA(A)* gene for rapeseed, *Glutamate dehydrogenase – GluD* gene for sugar beet, *alcohol dehydrogenase AdhC* gene for cotton and *phospholipase D* gene for rice (Jacchia et al., 2018). Laboratory L3 used the *alcohol dehydrogenase - ZmAdh1* gene as taxon-specific gene for maize rather than *hmgA* when testing the CRMs ERM®-BF412bk (Bt11), ERM®-BF423d (MIR604) and 1208-A (MIR162).

### 2.7 Digital PCR

Digital PCR analysis was performed on QX100 or QX200 TM droplet Digital PCR systems (Bio-Rad) according to the supplier's recommendations. The dPCR mixture contained 11 μL of dPCR Probe Supermix (Bio-Rad) used by L1, L3, L4 and dPCR Probe Supermix (no dUTP) (Bio-Rad) by L2, 1.1 μL of forward primers, 1.1 μL of reverse primer and 1.1 μL of probe. L1 and L4 added 7.7 μL of sample DNA at two concentration levels for a total PCR reaction volume of 22 μL corresponding to a concentration range varying between 21 and 245 ng/reaction and between 15.5 and 154 ng/reaction for L1 and L4, respectively. Lab 3 added 5 μL of DNA sample in a final volume of 20 μL at concentration levels ranging between 15.5 and 154 ng/reaction. L2 quantified the sample DNA with a spectrophotometer and the measured DNA concentration varying from 5 to 694 ng/μL could not be compared with the values determined by the other laboratories using a fluorimeter.

Twenty microliters of this mixture and 70 μL of droplet oil (Bio-Rad) were loaded in a microfluidic cartridge (Bio-Rad). Droplets were generated using the QX100 (L1, L3), QX200 (L2, L4) or AutoDG (L1) droplet generator (Bio-Rad) and the emulsion was transferred manually in a C1000 Touch thermal cycler (Bio-Rad) set at a ramping rate of 2 °C/s to ensures a uniform thermal transfer to all droplets. The general thermocycling conditions applied by the four laboratories were slightly different. The initial activation of the polymerase was identical (10 min at 95 °C), followed by 40 (L2, L3, L4) to 45 (L1) cycles consisting in a denaturation at 94 °C (L3) or 95 °C (L1, L2, L4) during 15 s (L2), 30 s (L3, L4) or 60 s (L1) and an annealing step at 60 °C for 1 min. The Taq polymerase was finally inactivated and the droplet shell hardened at 98 °C for 10 min.

### 2.8 Data reading and analysis

The read-out of droplets with positive and negative signals was performed with an auto fluorescence amplitude threshold setting with the combined wells option using the QuantaSoft software (version 1.7.4.0917) and the Bio-Rad droplet readers QX100 (L1) or QX200 (L2, L3, L4) because a clear segregation between the positive and negative droplets was observed. The target DNA concentration was calculated from the fraction of positive droplets (positive end-point reactions) and the number of accepted droplets using Poisson statistics taking into account dilution factors and droplet volumes of 0.834 nL and 0.786 nL for the dPCR Probe Supermix and the dPCR Probe Supermix (no UTP), respectively (Corbisier et al., 2015; Emslie et al., 2019). The droplet volumes have nevertheless no impact on the determination of the conversion factors, because the latter are calculated as a ratio of the GM target DNA concentration divided by the taxon-specific DNA concentration, both determined using the same mastermixes.

### 2.9 Exclusion criteria for the data sets

Measurement results from single PCR wells containing the droplets were excluded on the basis of technical reasons in case any of the following conditions was met: (i) the total number of accepted droplets was <10,000 for individual results; (ii) *adh1* rather than *hmg* was used as taxon-specific target for maize; (iii) the average fluorescence amplitudes of positive or negative droplets were clearly different from those of the other wells on the plate; (iv) 5% of the accepted droplets had a fluorescence amplitude significantly below the average amplitude of the negative droplet cluster (i.e. < average – (4 × standard deviation)); (v) a construct-specific method was used in contrast to an event-specific one. Specific laboratory results were also excluded, if the relative standard deviation (RSD) of 6 replicate measurement results exceeded 20%.

The average number of accepted droplets of the valid measurement results was around 15500.

### 2.10 Resolution of the dPCR signals

The resolution measures how well the two populations (positive and negative droplets) can be differentiated. It is defined as the difference in fluorescence between the two peaks of the intensity curves for each population divided by the combined widths of the peaks. Resolutions were calculated directly from the dPCR fluorescence measurements using the R algorithm (Cloudy-V2-2.05) described earlier (Lievens, Jacchia, Kagkli, Savini, & Querci, 2016). The resolutions of the dPCR signals obtained by the 4 laboratories for each dPCR assay has been calculated and used to determine the quality of the generated data. Hence, a low resolution *per se* was not used as an exclusion criterion.

### 2.11 Calculation of the DNA copy number ratio

The DNA copy number ratio in one extract or in a gDNA solution was calculated for each laboratory as the ratio between the average of the reported transgene DNA copy numbers (*cp*_*T,i*_) to the average of the reported taxon-specific DNA copy numbers (*cp*_*E,j*_) with *i* and *j* being the number of replicates per extract or DNA solution tested for the transgenic and taxon-specific targets, respectively.

(1) $$\frac{cp_{T}}{cp_{E}}=\frac{\frac{\sum_{1}^{i}cp_{T,\;i}}{i}}{\frac{\sum_{1}^{j}cp_{E,\;j}}{j}}$$

For the powder CRMs, the average of the copy number ratios from the three extractions was calculated per laboratory and the overall DNA copy number ratio was averaged again among the laboratories.

The relative standard uncertainty of the overall copy number ratio (*u*_*cpr,%*_) was estimated using ANOVA. The calculations were performed with R (version 3.5.1) taking into account the within-lab and between-lab standard deviations (Equation (2)). For the liquid CRMs the standard error was used to assess u_cpr,%_.

(2) $$u_{cpr,\;\%}=\sqrt{\frac{MS_{w}+\left(\frac{MS_{b}-MS_{w}}{n-1}\right)}{n}}\;\times \;100$$

*MS*_*w*_: Mean square within lab. *MS*_*b*_: Mean square between lab. *n*: number of replicates.

### 2.12 Calculation of the conversion factor (*CF*_*CRM*_)

The GM mass fraction (*w*_*CRM*_) of a CRM is the mass (in g) of the GM material divided by the total mass (in g) of the GM and non-GM material. It is provided as certified value and commonly expressed in g/kg for a powder CRM according to Equation (3).

(3) $$w_{CRM}=\frac{m_{GM}\;}{m_{GM}+m_{nonGM}}\times 1000$$

A *CF*_*CRM*_ can therefore be calculated as:

(4) $$CF_{CRM}=\frac{cp_{T\;}}{cp_{E}}\;\times \;\frac{1}{w_{CRM}}\;\times 1000$$

where cp_T_ is representing the copy number of the transgenic sequence and cp_E_ the copy number of the taxon-specific sequence.

For CRMs composed of solutions of purified gDNA extracted from GM leaf materials, the certified values are provided on the certificates in ng/μg, but the same Equation (4) can be applied. The impurities in the DNA solution that could count as non-GM is often neglected. For GM CRMs certified as ‘pure’, the mass of the non-GM powder was considered as being zero and the mass fraction of the GM has been taken as 1000 g/kg. For CRMs certified to contain more than a certain GM mass fraction (e.g. > 986 g/kg), the mean between the upper and lower limits was taken as the GM mass fraction (in our example: (1000 + 986)/2 = 993 g/kg).

The conversion factor was calculated applying Equation (4), while the associated relative standard uncertainty of the conversion factor (*u*_*cf,%*_) has been derived from the relative standard uncertainty of the overall copy number ratio (*u*_*cpr,%*_) and the relative standard uncertainty of the certified CRM property value (*u*_*CRM,%*_):

(5) $$u_{cf,\%}=\sqrt{u_{cpr\%}^{2}+u_{CRM\%}^{2}}$$

The conversion factors (CF_CRM_) for 50 CRMs, listed in Table 2, were calculated as an average of technically acceptable data sets provided by at least 2 laboratories. A maximum of 24 data points and a minimum of 16 data points for both transgenic and taxon-specific targets have been used to calculate the ratio in each CRM consisting of a solution of gDNA. For each powder CRM, the corresponding CF_CRM_ was calculated as the ratio of up to 72 data points for the transgenic as well as for the taxon-specific targets (see details in Table S1.1) and based on up to 22 data points for the CRMs consisting of extracted genomic DNA solutions (see details in Table S1.2).

**Table 2:** Conversion factor (CF_CRM_) and associated expanded uncertainty (U_CF_) determined for GM CRMs listed per taxon. The coding of the transgenic and taxon-specific targets is as referenced in the EURL GMFF method database for qPCR methods.

CRM code	Event	EU reference method	taxon-specific target	CF_CRM_	U_CF_ (*k* = 2)
GM maize					
ERM®-BF412bk	Bt11	QT-EVE-ZM-015	hmgA QT-TAX-ZM-002	0.37	0.03
ERM®-BF424d	DAS59122	QT-EVE-ZM-012		0.34	0.05
ERM®-BF418d	1507	QT-EVE-ZM-010		0.61	0.09
0407-B	GA21	QT-EVE-ZM-007		0.35	0.06
ERM®-BF415f	NK 603	QT-EVE-ZM-014 QT-EVE-ZM-008		0.51	0.04
0406-D	MON88017	QT-EVE-ZM-016		0.54	0.06
0906-E	MON89034	ZM-QT-EVE-018		0.58	0.04
ERM®-BF423d	MIR604	QT-EVE-ZM-013		0.45	0.03
1208-A2	MIR162	QT-EVE-ZM-022		0.58	0.06
0709-A	MON 87460	QT-EVE-ZM-005		0.38	0.10
0512-A	MON 87427	QT-EVE-ZM-003		0.58	0.05
ERM®-BF433d	DAS-4Ø278-9	QT-EVE-ZM-004		0.36	0.05
ERM®-BF420c	3272	QT-EVE-ZM-019		0.42	0.07
0411-D	5307	QT-EVE-ZM-002		0.36	0.05
ERM®-BF438e	VCO01981	QT-EVE-ZM-001		0.48	0.05
ERM®-BF411f	Bt176	QT-EVE-ZM-023		0.68	0.05
ERM®-BF416d	MON863	QT-EVE-ZM-009		0.62	0.08
GM rice					
0306-I8	LLRICE62	QT-EVE-OS-002	Phospholipase D - PLD QT-TAX-OS-017	0.82	0.15
GM Cotton					
0113-A	MON 88701	QT-EVE-GH-010	alcohol dehydrogenase - AdhC QT-TAX-GH-018	1.10	0.11
0804-B	MON1445	QT-EVE-GH-003		1.05	0.08
0804-D	MON15985	QT-EVE-GH-005		0.96	0.08
0804-C	MON 531	QT-EVE-GH-004		0.99	0.11
0306-E2	LLCotton25	QT-EVE-GH-002		1.00	0.08
1108-A5	GHB614	QT-EVE-GH-006		1.11	0.06
ERM®-BF422d	281-24-236	QT-EVE-GH-001a		0.99	0.17
ERM®-BF422d	3006-210-23	QT-EVE-GH-001b		1.02	0.18
ERM®-BF429c	T304-40	QT-EVE-GH-009		1.27	0.16
0906-D	MON 88913	QT-EVE-GH-007		1.02	0.07
GM soybean					
ERM®-BF432d	DAS-68416-4	QT-EVE-GM-013	lectin - Le1 QT-TAX-GM-002 or QT-TAX-GM-009	1.17	0.19
ERM®-BF436e	DAS-44406-6	QT-EVE-GM-015		0.99	0.13
ERM®-BF437e	DAS-81419-2	QT-EVE-GM-014		0.85	0.10
0707-B10	A2704-12	QT-EVE-GM-004		0.97	0.03
0906-B	MON89788	QT-EVE-GM-006		0.98	0.07
ERM®-BF410ep	40-3-2	QT-EVE-GM-005		0.79	0.14
0809-A	MON87701	QT-EVE-GM-010		0.95	0.05
ERM®-BF425d	356043	QT-EVE-GM-009		0.98	0.14
0707-C6	A5547-127	QT-EVE-GM-007		1.01	0.07
0210-A	MON 87705	QT-EVE-GM-005		0.96	0.07
0311-A	MON 87708	QT-EVE-GM-012		1.00	0.10
0809-B	MON 87769	QT-EVE-GM-002		0.99	0.06
ERM-BF426d	305423	QT-EVE-GM-008		0.93	0.11
0911-D	BPS-CV127-9	QT-EVE-GM-011		1.01	0.11
0610-A4	FG 72	QT-EVE-GM-001		1.03	0.07
GM swede-rape					
0306-F6	MS8	QT-EVE-BN-002	Acyl-[acyl-carrier-protein] hydrolase - FatA(A) QT-TAX-BN-001	050	0.05
0306-G5	Rf3	QT-EVE-BN-003		1.01	0.10
GM oilseed rape					
ERM®-BF434e	73496	QT-EVE-BN-009	Acyl-[acyl-carrier-protein] hydrolase - FatA(A) QT-TAX-BN-001	0.95	0.18
0304-B2	GT73	QT-EVE-BN-004		0.92	0.10
0208-A5	T45	QT-EVE-BN-001		0.95	0.04
1011-A	MON 88302	QT-EVE-BN-010		0.96	0.04
GM sugar beet					
ERM®-BF419b	H71	Glutamine synthase - GS2 QT-TAX-BV-013	Glutamine synthase - GS2 QT-TAX-BV-013	0.48	0.05

## 3 Results

### 3.1 Transferability of real-time qPCR procedures to digital PCR procedures

The four laboratories involved in this study were asked to use the primers and probe oligo sequences published by the EURL GMFF for the quantification of the GM content by real-time qPCR methods. The quantity of DNA added in the dPCR procedure, the final concentration of the primers and probe, the type of fluorophore, the nature of the quencher, the annealing temperature during the PCR and the number of PCR cycles have been adapted for each of the 52 dPCR methods targeting GM events and the 7 dPCR methods targeting taxon-specific reference genes (see details in Tables S1.3–6).

The DNA solutions/extracts were diluted to achieve a ratio of positive droplets to total droplets of around 0.8. At that ratio, the uncertainty related to the conversion into a number of DNA copies per volume is the lowest (Pinheiro et al., 2012). The four laboratories were asked to perform three DNA extractions and have tested each extract at slightly different concentrations of dsDNA. L1 used 112 and 224 ng of DNA for the maize measurements, 60 and 120 ng of DNA for the cotton measurements, 28 and 56 ng of DNA for the soya measurements, 29 and 58 ng of DNA for the rapeseed measurements, 21 and 42 ng of DNA for the rice measurements, and 29 and 58 ng for the sugar beet measurements. L3 has used 33.5 and 100 ng of DNA for the maize and cotton tests, 16.5 and 50 ng of DNA for the soya and rapeseed tests, 6 and 18.5 ng of DNA for the rice tests, and 10.5 and 31 ng of DNA for the sugar beet tests. L2 also adapted the DNA concentration in function of the genome length of the species tested, but did not quantify in contrast to the other labs the dsDNA in solution and estimated the total DNA concentration by UV spectrometry. Therefore, the DNA concentration measured by L2 varied from 1 to 250 ng of DNA depending on the CRM tested.

No particular preference can be given regarding an optimal extraction method for the extraction of DNA from CRMs. The laboratories used indeed different extraction methods (CTAB-based method followed or not by an additional purification steps on Tip20 column and several commercial kits (Nucleospin Food kit, a Foodproof GMO Sample Preparation kit III, Maxwell®RSC Pure Food GMO and Authentication kit) to extract the DNA from the powder CRMs and obtained very similar results in terms of variability (RSD) of their dPCR results even if some differences among the different methods in terms of yield (in ng DNA/mg powder) were observed (data not shown here). The extraction of DNA from CRMs being by definition homogeneous and by nature a relative easy matrix composed of pure ground seeds did not represent a particular challenge in this study.

The four laboratories used different amplicons within the lectin *Le1* gene for the taxon-specific soybean method. This lack of a consensus is remarkable but did not introduce a noticeable variability for the quantification of GM soybean events. As laboratories were invited to use their preferred dPCR method to measure the transgene/taxon-specific gene copy number ratio, different experimental conditions have been used. However, there were only small differences between the experimental conditions chosen by the laboratories, which concerned mainly the final concentrations for the primers and probe (varying e.g. from 50 nM to 900 nM for the forward primer in the maize 3272 procedure) or different fluorescent quenchers or probe/quencher combinations for the same probe sequence (e.g. for MON87701 procedures: FAM/TAMRA, FAM/BHQ1, HEX/BHQ1). The replacement of dual labelled hydrolysis probes (validated for the qPCR methods) by non-fluorescent quenchers such as BHQ1 or MGBNFQ and MGBEQ for the dPCR methods has also been successfully used in this study. Non-fluorescent quenchers reduce the background fluorescence and had been suggested to improve the signal-to-noise ratio in dPCR measurements (Dobnik et al., 2015). Minor grove binding (MGB) quenchers can form a stable duplex, which is increasing the melting temperature of the probe and are therefore often used to allow shorter amplicons.

### 3.2 Reduction of the ‘rain’ signals

**Fig. 2:**
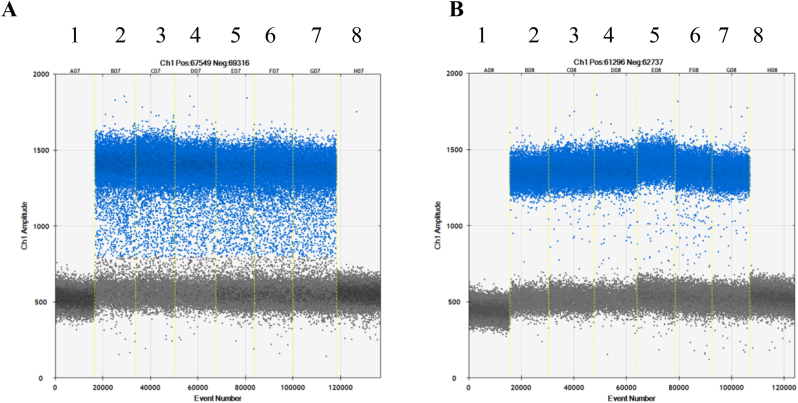
Example of the reduction of the rain signals in dPCR measurements of cotton MON531 (A) before and (B) after fragmentation of the extracted genomic DNA by enzymatic restriction with *Mse*I. Lanes 2–4 with 60 ng of gDNA and lanes 5–8 with 120 ng of gDNA per PCR reaction. Lanes 1 and 8 are negative controls (NTC).

The fragmentation of gDNA prior to partitioning is suggested by the Bio-Rad manufacturer to reduce the viscosity of the DNA, thereby facilitating a homogenous distribution of the DNA targets across the droplets and generating droplets of the same size (Kaihara, Bemis, & Choudhary, 2016). A restriction of the DNA extracted from plant samples for concentrations above 66 ng per 20 μL of reaction volume is also recommended by the ENGL (Pecoraro et al., 2019). L1 systematically restricted the gDNA when rain was observed in some dPCR measurements. A clear reduction of the rain could be observed in some cases, as illustrated with the MON531 cotton dPCR measurements on unrestricted and restricted gDNA, respectively (Fig. 2). The benefit of analysing fragmented DNA has been reported earlier (Grelewska-Nowotko, Żurawska-Zajfert, Żmijewska, & Sowa, 2018). However, robust PCR methods do not require a prior fragmentation of the gDNA. The advantage of testing restricted DNA cannot be generalised, has to be explored on a case-by-case basis and is still controversial. Indeed, while enzymatic restriction of human genomic DNA increases accessibility for some assays, in well-optimised PCR assays it can reduce the number of amplifiable targets and increase assay variability relative to the uncut sample (Kline, Romsos, & Duewer, 2016).

### 3.3 Optimised annealing temperatures

**Fig. 3:**
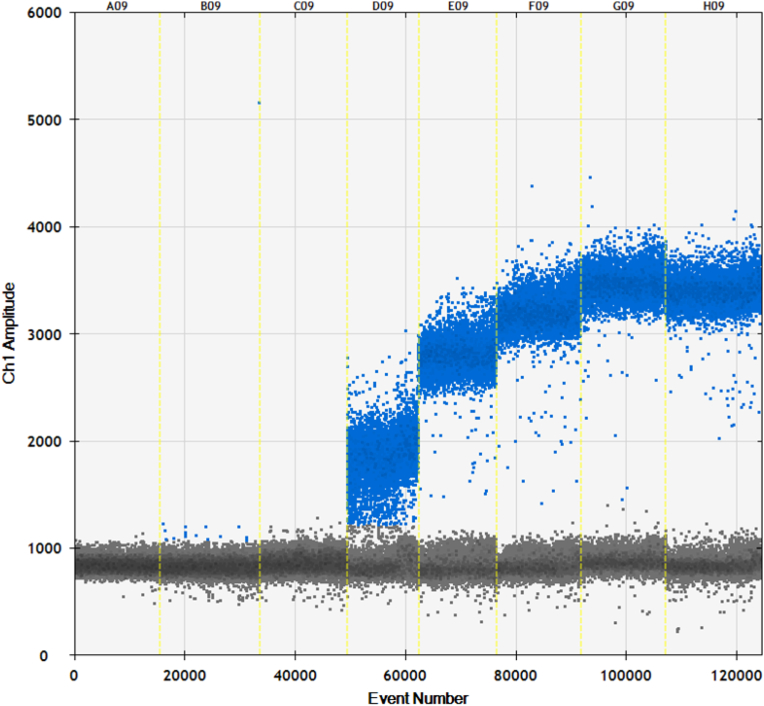
Example for an improved resolution by lowering the annealing temperature for dPCR measurements of MON88913. Lane A (65 °C), Lane B (64.1 °C), Lane C (62.1 °C), Lane D (59.3 °C), Lane E (55.9 °C), Lane F (53 °C), Lane G (51 °C), Lane H (50 °C).

Several thermocycling conditions for the digital PCR have been optimised by Labs 1, 3 and 4. An annealing temperature of 60 °C has been recommended in the qPCR methods validated by the EURL GMFF. In a few cases, a poor resolution of the negative and positive peaks was observed with an annealing temperature of 60 °C. A better resolution was obtained for some dPCR methods by lowering the annealing temperature as illustrated in Fig. 3. L1 has reduced the annealing temperature to 50 °C for measuring the GM events MON531 and MON88913, to 53 °C for GM event MON87427, and to 55 °C for GM events Bt11, 1507, MON88017, MON89034, MIR604, MIR162, MON87460, 5307, LLCotton25, MON88701, MON1445, 3006-210-23, FG72, MON88302 and H71. The other GM events where tested with an annealing temperature of 60 °C. L2 used 60 °C for all dPCR measurements, whereas L3 changed the annealing temperature to 55 °C for the event MON531 and to 57 °C for the GM events MON88302 and H71. L4 also changed the annealing temperature to 55 °C for measuring GM events Bt11, 1507, MON88017, LLCotton25 and 3006-210-23. The lowering of the annealing temperatures did not result in any unspecific amplification.

### 3.4 Assessment of the quality of the dPCR measurement results

A general assessment of the quality of the dPCR measurement results generated by the four laboratories has been made using three evaluation criteria, namely (i) the percentage of rain, (ii) the resolution and (iii) the RSD among data from replicate measurements. Laboratories 1, 2 and 3 tested 52 GM events, whereas laboratory 4 was only asked to analyse 18 GM events. This general assessment allowed to discriminate between specific problems with a CRM and the sub-optimal performance of a transferred dPCR method within a particular laboratory. Arbitrary criteria have been chosen a posteriori to define what could be considered as good, borderline or unsatisfactory outcome for the three evaluation criteria. A percentage of rain below 1% was considered as good, between 1 and 2.5% as acceptable and above 2.5% as unsatisfactory. A resolution above 2.5 was considered as good, between 1.5 and 2.5 as borderline and below 1.5 as unsatisfactory. A RSD below 5% was considered as excellent, below 10% as good, between 10 and 15% as borderline and above 15% as unsatisfactory. In terms of rain, laboratory 3 was the only lab where the measurement results of 3 out of 52 dPCR methods obtained an unsatisfactory score. The defined parameters (amount of rain, resolution and RSD) and observed limits can be used by a single laboratory as minimum performance characteristics to optimise the performance of their dPCR methods.

## 4 Discussion

In most of the cases the qPCR methods could be easily transferred into dPCR methods by using the same oligo sequences and final oligo concentration. Laboratories have nevertheless used different strategies to improve the resolution or to reduce the rain in their dPCR measurements.

Obviously, some qPCR methods are easily transferable into a dPCR format than others, but this study did not identify general forecasting rules. Some interesting findings concerned the need to use common taxon-specific targets to normalise the GM content measured by dPCR. Two maize-specific reference methods targeting different chromosomal genes, encoding for the high mobility group I protein (*hmg)* and the alcohol dehydrogenase (Zm*adh*1), both present as single copy genes in the maize genome have been investigated with dPCR. Both targets are commonly used to normalise the measurement results for GM maize events obtained by qPCR. Here they were tested on genomic DNA extracted from different maize CRMs (Bt11, MIR604, MIR162). For the *hmg* measurements with dPCR, a 79 bp fragment was amplified by L1, L2 and L4, whereas a longer amplicon (135 bp) was amplified in the Zm*adh*1 measurements by L3. The probes for both methods have been labelled with the same fluorescent marker and quencher, 6-FAM and TAMRA, respectively. Consistent copy number ratios were observed independently of the maize reference target tested for the MIR604 and MIR162 methods by the 4 laboratories. However, the Bt11/Zm*adh*1 copy number ratio reported by L3 was significantly higher (*p* value < 0.05) than the Bt11/hmg ratio, namely 0.42 ± 0.02 and 0.37 ± 0.03, respectively. As the number of Bt11 targets is the same in the CRM ERM-BF412k used by the four laboratories, it seems that more copies of *hmg* than copies of Zm*adh1* were quantified by dPCR. This result is in line with observations made earlier, where 13% more copies of *hmg* than copies of Zm*adh*1were detected by dPCR (Jacchia et al., 2018). However, with other combinations of methods such as MIR604/Zm*adh1* or MIR162/Zm*adh*1 no significant difference was observed compared to the MIR604/hmg or MIR162/hmg ratios. These observations underline the recommendation to design PCR methods with amplicons of similar size for the numerator and the denominator of the ratio calculation (Debode, Marien, Janssen, Bragard, & Berben, 2017). The pair Bt11 (68 bp)/*Zmadh*1 (135 bp) combines the smallest amplicon length for the GM event and the longest amplicon length for the maize reference gene. This slightly unbalanced combination of amplicon lengths may explain the apparent lack of robustness of the Bt11/Zm*adh*1 methods when it is transferred into a dPCR format and the significant bias observed by L3. This study confirms that *hmg* is the most reliable reference target to quantify maize GM which corroborates earlier studies performed with qPCR (Paternò et al., 2009) and dPCR (Jacchia et al., 2018). Hence, the CF_CRM_ for maize reported in Table 2 were all normalised using the *hmg* method.

Significantly different conversion factors were also obtained for the ERM®-BF411f maize CRM containing about 50 g/kg of Bt176 maize depending on the Bt176 target used. L2 used a construct-specific method targeting a gene fragment coding for the synthetic CryIA(b) protein (ISO, 2005), whereas L1, L3 and L4 used an event-specific method targeting an 82-bp fragment of the integration region of the construct inserted into the maize plant genome at the 3′ flanking region (referenced as QT-EVE-ZM-023). Hence, the CF_ERM®-BF411f_ was calculated using only the dPCR results from L1, L3 and L4.

The CF_CRM_ listed in Table 2 can be clustered into 3 classes that are related to the theoretical ratios of transgenic and taxon-specific targets in the plant material used to produce the CRM. For CRMs made of homozygous plant material (soybean, rapeseed, cotton, rice) or of gDNA extracted from leaves a theoretical ratio of 1 is expected. For the 15 soybean CRMs tested, the CF_CRM_ varied from 0.79 ± 0.14 (ERM-BF-410ep event 40-3-2) to 1.17 ± 0.19 (ERM-BF-432d event DAS-68416-4) with an overall average CF_CRM_ of 0.97 for all tested soybean CRMs. For the 10 cotton CRMs tested, the CF_CRM_ varied from 0.96 ± 0.08 (0804-D event MON15985) to 1.27 ± 0.16 (ERM-BF-429c event T304-40) with an average CF_CRM_ of 1.05. Only 4 rapeseed CRMs were analysed, the CF_CRM_ varied from 0.92 ± 0.10 (0304-B2 event GT73) to 0.96 ± 0.04 (1011-A event MON88302) with an average CF_CRM_ of 0.95. The sole rice CRM tested (0306-I8 event LLRice62) had a CF_CRM_ of 0.82 ± 0.15. Moreover, a homozygous swede rape CRM (0306-G5 event Rf3) had a CF_CRM_ of 1.01 ± 0.06.

For maize CRMs made of pure hemizygous seed material, the ratio will depend upon a number of factors such as the origin of the transgenic donor and the proportion of DNA that could be extracted from the endosperm of the seeds (Zhang, Corlet, & Fouilloux, 2008). A copy number ratio may theoretically vary between 0.33 and 0.5 when the transgenic donor is a male, and between 0.5 and 0.67 when the transgenic donor is a female. If the endosperm represents 50% of the total DNA extracted, a conversion factor of 0.43 or 0.59 has been predicted for a pure material composed of GM maize with a male or female transgenic donor, respectively.

In this study, the CF_CRM_ measured in hemizygous maize CRMs from a male transgenic donor varied from 0.34 ± 0.05 for ERM®-BF424d (event DAS59122) to 0.68 ± 0.05 for ERM®-BF411f (event Bt176), whereas the values ranged from 0.51 ± 0.04 (for ERM®-BF415f event NK603) to 0.62 ± 0.08 (ERM®-BF416d event MON863) in hemizygous maize CRMs with a female transgenic donor.

The unexpected high CF_CRM_ (0.68 ± 0.05) observed for ERM®-BF411f certified to contain about 50 g/kg of Bt176 maize questions the breeding information obtained from the GM producer. The breeding information on the certificate mentions a male transgenic donor and similar amounts of DNA could be extracted from the Bt176 maize powder and the non-GM counterpart (Trapmann et al., 2004).

The two maize CRMs MON87427 and MON89034, for which no information about the parental origin of the transgenic donor is mentioned on the respective certificates, had been probably made of female transgenic donors, because the obtained CF_CRM_ of 0.58 ± 0.05 and 0.58 ± 0.04, respectively, are above 0.5 (Table 2). The parental origin of the transgenic donor for the MON87460 material is most probably male, as the CF_CRM_ assigned has a value of 0.38 ± 0.1, whereas no firm conclusion can be made for CRM MON88017 as the assigned CF_CRM_ of 0.54 ± 0.06 is close to 0.5.

For the sugar beet CRM (ERM-BF419b event H71) and the hemizygous swede rape (0306-F6 event Ms8) the measured CF_CRM_ were 0.48 ± 0.05 and 0.50 ± 0.03, respectively.

The conversion of dPCR results into mass fraction data with a common CF_CRM_ of 1.0 for a GM soybean rather than using the specific CF_CRM_ would cause a positive bias of up to 15% for the quantification of the event DAS-81419-2 and a negative bias of 17% for the quantification of the event DAS-68416-4. The pragmatic approach followed up to now by the EURL GMFF to recommend a CF of 1 for the conversion of measurement results obtained as DNA copy number ratios into mass fractions for soybean events seemed to be reasonable. The CF_CRM_ determined for the CRM ERM-BF410ep (event 40-3-2, nominal relative mass fraction 10%) is abnormally low (0.79 ± 0.14) for a homozygous material. However, this CRM is composed of a mixture of GM and non-GM soybean powders that may have different DNA contents. The ratio of the DNA masses extractable from 100 mg of GM and non-GM powders, respectively, was 0.761 ± 0.011 (Dimitrievska et al., 2017) which explains the CF measured by dPCR here.

The conversion factors measured for the cotton species are around 1, with some deviations observed for A113-A (event MON 88701), 1108-A5 (event GHB614) and ERM-BF429c (event T304-40) having CF_CRM_ of 1.10 ± 0.11, 1.11 ± 0.06 and 1.27 ± 0.16, respectively.

We therefore recommend to use the specific CF_CRM_ published in this paper for all conversions of copy number ratios into mass fractions, independently of the zygosity of the CRM.

No agreement could be found for the measurement results from the four laboratories for the conversion factors of cotton event BCS-GHØØ5-8 (ERM®-BF428c) and maize event T25 (0306-H9). Both CRMs need to be further examined.

Fifteen other CRMs (0208-A6, 0306-F7, 0306-G6, 0711-A3, 0711-B2, 0711-C3, 0711-D4, ERM®-BF440e, 0306-I9, 0215-A, 0215-B, 0216-A, ERM®-BF411gk, ERM®-BF413gk, ERM®-BF439e), which were not part of this study, have been investigated later in a follow up study and the respective CF_CRM_ are published via the website of the EURL GMFF (EURL GMFF, 2021b).

## 5 Conclusion

In this study, the ratios of the numbers of transgenic target sequences vs the numbers of taxon-specific reference target sequences in 50 GMO CRMs have been determined by independent laboratories with small uncertainties. Therefore, measurement results obtained by dPCR on unknown samples and expressed in DNA copy number ratios can now be converted in a harmonised manner into mass fraction data with an agreed common and traceable CF_CRM_ as recommended previously (Corbisier & Emons, 2019). The uncertainty associated with the conversion factor has always to be included into the uncertainty of the converted result.

The measurement system for official GMO controls in the EU is composed of qPCR methods validated by the EURL GMFF according to ISO/IEC 17025 and specified criteria and of CRMs produced according to ISO 17034. The dPCR methods described in this paper and the reference list of conversion factors per CRM can be applied to test unknown samples by dPCR and to convert the measurement result into a mass fraction as required by EU legislation.

## CRediT authorship contribution statement

**Philippe Corbisier:** Conceptualization, Writing – original draft, and reviewing. **Gerhard Buttinger:** Investigation, Validation, Formal analysis, reviewing. **Cristian Savini:** Investigation. **Maria Grazia Sacco:** Investigation. **Francesco Gatto:** Investigation, reviewing. **Hendrik Emons:** Writing – review & editing.

## Declaration of competing interest

The authors declare that they have no known competing financial interests or personal relationships that could have appeared to influence the work reported in this paper.
