# Supplementary material for: Expression of GM content in mass fraction from digital PCR data
Source: Food Control. 2022 Mar;133(Pt B):108626. doi: 10.1016/j.foodcont.2021.108626 (PMC8756621; doi:10.1016/j.foodcont.2021.108626)
Supplement: Multimedia component 1 [file mmc1.docx]

**Expression of GM content in mass fraction from digital PCR data**

Philippe Corbisier, Gerhard Buttinger, Cristian Savini, Maria Grazia Sacco, Francesco Gatto, Hendrik Emons

**List of Supplementary materials**

**Table S1.1:** Detailed data providing the average copy number concentrations (cp/µL) per extract, per laboratory for both endogen and taxon-specific assays as well as the calculated endogen/taxon-specific ratio, mean ratio per lab and overall mean ratio used for the determination of the conversion factors of powder CRMs.

**Table S1.2:** Detailed data providing the average copy number concentrations (cp/µL) per laboratory for both endogen and taxon-specific assays as well as the calculated endogen/taxon-specific ratio, mean ratio per lab and overall mean ratio used for the determination of the conversion factors in CRM provided as genomic DNA solutions.

**Table S1.3:** Oligonucleotides used as targets for the ddPCR taxon-specific methods. A fluorescent marker, quencher or oligo sequence coloured in red indicates a difference with the qPCR method published in the EURL GMFF method database.

**Table S1.4:** Oligonucleotides used as targets for the ddPCR GM maize methods. A fluorescent marker, quencher or oligo sequence coloured in red indicates a difference with the qPCR method published in the EURL GMFF method database.

**Table S1.5**: Oligonucleotides used as targets for the ddPCR GM cotton methods. A fluorescent marker, quencher or oligo sequence coloured in red indicates a difference with the qPCR method published in the EURL GMFF method database.

**Table S1.6:** Oligonucleotides used as targets for the ddPCR GM soybean methods. A fluorescent marker, quencher or oligo sequence coloured in red indicates a difference with the qPCR method published in the EURL GMFF method database.

**Table S1.7:** Oligonucleotides used as targets for the ddPCR GM rapeseed, sugarbeet and rice methods. *methods*. A fluorescent marker, quencher or oligo sequence coloured in red indicates a difference with the qPCR method published in the EURL GMFF method database.

**Table S1.8:** Verification list for Minimum Information for Publication of Quantitative Digital PCR Experiments (dMIQE)

**Table S1.1:** Detailed data providing the average copy number concentrations (cp/µL) per extract, per laboratory for both endogen and taxon-specific assays as well as the calculated endogen/taxon-specific ratio, mean ratio per lab and overall mean ratio used for the determination of the conversion factors in powder CRMs.

| **CRM** | **Lab** | **Extract** | **transgen** | **Replicates transgen** | **Taxon** | **Replicates Taxon** | **ratio** | **mean lab ratio** | **overall mean ratio** |
| --- | --- | --- | --- | --- | --- | --- | --- | --- | --- |
|  |  |  | [cp/μl] |  | [cp/μl] |  |  |  |  |
| ERM®-BF412bk | **Lab1** | **1** | **30146** | **6** | **11025** | **6** | **0,37** | **0,358** | 0,36 |
| maize |  | **2** | **11119** | **6** | **3930** | **6** | **0,35** |  |  |
|  |  | **3** | **14017** | **6** | **4978** | **6** | **0,36** |  |  |
|  | Lab2 | 1 | 9002 | 6 | 3555 | 6 | 0,39 | 0,363 |  |
|  |  | 2 | 7990 | 6 | 2923 | 6 | 0,37 |  |  |
|  |  | 3 | 7011 | 6 | 2305 | 6 | 0,33 |  |  |
|  | **Lab4** | **1** | **59942** | **6** | **21497** | **6** | **0,36** | **0,370** |  |
|  |  | **2** | **77324** | **6** | **29018** | **6** | **0,38** |  |  |
|  |  | **3** | **87918** | **6** | **33186** | **6** | **0,38** |  |  |
| ERM®-BF424d | Lab2 | 1 | 6698 | 6 | 242 | 6 | 0,04 | 0,036 | 0,03 |
| maize |  | 2 | 6597 | 6 | 237 | 6 | 0,04 |  |  |
|  |  | 3 | 7184 | 6 | 251 | 6 | 0,03 |  |  |
|  | **Lab3** | **1** | **5712** | **6** | **179** | **6** | **0,03** | **0,033** |  |
|  |  | **2** | **5713** | **6** | **192** | **6** | **0,03** |  |  |
|  |  | **3** | **5663** | **6** | **189** | **6** | **0,03** |  |  |
|  | Lab4 | 1 | 60266 | 6 | 1999 | 6 | 0,03 | 0,033 |  |
|  |  | 2 | 48729 | 6 | 1522 | 6 | 0,03 |  |  |
|  |  | 3 | 53701 | 6 | 1875 | 6 | 0,03 |  |  |
| ERM®-BF418d | **Lab1** | **1** | **22711** | **6** | **1305** | **6** | **0,06** | **0,056** | 0,06 |
| maize |  | **2** | **7880** | **6** | **447** | **6** | **0,06** |  |  |
|  |  | **3** | **9014** | **6** | **489** | **6** | **0,05** |  |  |
|  | Lab3 | 1 | 6050 | 6 | 345 | 6 | 0,06 | 0,059 |  |
|  |  | 2 | 5607 | 6 | 314 | 6 | 0,06 |  |  |
|  |  | 3 | 5120 | 6 | 323 | 6 | 0,06 |  |  |
|  | **Lab4** | **1** | **58500** | **6** | **3431** | **6** | **0,06** | **0,065** |  |
|  |  | **2** | **73835** | **6** | **4467** | **6** | **0,06** |  |  |
|  |  | **3** | **65094** | **4** | **4915** | **6** | **0,08** |  |  |
|  |  |  |  |  |  |  |  |  |  |
|  |  |  |  |  |  |  |  |  |  |
| 0407-B | Lab1 | 1 | 41099 | 6 | 12014 | 6 | 0,29 | 0,296 | 0,35 |
| maize |  | 2 | 43884 | 6 | 13257 | 6 | 0,30 |  |  |
|  |  | 3 | 39231 | 6 | 11550 | 6 | 0,29 |  |  |
|  | **Lab2** | **1** | **13078** | **6** | **4997** | **6** | **0,38** | **0,375** |  |
|  |  | **2** | **9921** | **6** | **3611** | **6** | **0,36** |  |  |
|  |  | **3** | **9915** | **6** | **3772** | **6** | **0,38** |  |  |
|  | Lab3 | 1 | 11188 | 6 | 4031 | 6 | 0,36 | 0,374 |  |
|  |  | 2 | 10743 | 6 | 4046 | 6 | 0,38 |  |  |
|  |  | 3 | 11328 | 6 | 4351 | 6 | 0,38 |  |  |
|  | **Lab4** | **1** | **78999** | **5** | **27176** | **6** | **0,34** | **0,355** |  |
|  |  | **2** | **55425** | **5** | **19939** | **6** | **0,36** |  |  |
|  |  | **3** | **77953** | **6** | **28111** | **6** | **0,36** |  |  |
| ERM®-BF415f | Lab1 | 1 | 28352 | 5 | 738 | 5 | 0,03 | 0,026 | 0,02 |
| maize |  | 2 | 2949 | 6 | 76 | 6 | 0,03 |  |  |
|  |  | 3 | 16685 | 6 | 447 | 6 | 0,03 |  |  |
|  | **Lab2** | **1** | **5822** | **6** | **150** | **6** | **0,03** | **0,024** |  |
|  |  | **2** | **5566** | **6** | **124** | **6** | **0,02** |  |  |
|  |  | **3** | **5599** | **6** | **138** | **6** | **0,02** |  |  |
|  | Lab3 | 1 | 5555 | 6 | 136 | 6 | 0,02 | 0,025 |  |
|  |  | 2 | 5269 | 6 | 132 | 6 | 0,02 |  |  |
|  |  | 3 | 6064 | 6 | 146 | 6 | 0,02 |  |  |
| 0406-D | **Lab2** | **1** | **9449** | **6** | **4853** | **6** | **0,51** | **0,521** | 0,54 |
| maize |  | **2** | **9396** | **6** | **4925** | **6** | **0,52** |  |  |
|  |  | **3** | **8703** | **6** | **4581** | **6** | **0,53** |  |  |
|  | Lab3 | 1 | 2768 | 6 | 1548 | 6 | 0,56 | 0,564 |  |
|  |  | 2 | 3018 | 6 | 1718 | 6 | 0,57 |  |  |
|  |  | 3 | 3236 | 6 | 1827 | 6 | 0,56 |  |  |
|  | **Lab4** | **1** | **21789** | **6** | **11213** | **6** | **0,51** | **0,520** |  |
|  |  | **2** | **14110** | **6** | **7399** | **6** | **0,52** |  |  |
|  |  | **3** | **13455** | **6** | **7023** | **6** | **0,52** |  |  |
| 0906-E | Lab1 | 1 | 34458 | 6 | 20692 | 6 | 0,60 | 0,582 | 0,58 |
| maize |  | 2 | 39324 | 6 | 21981 | 6 | 0,56 |  |  |
|  |  | 3 | 39023 | 6 | 22908 | 6 | 0,59 |  |  |
|  | **Lab2** | **1** | **11333** | **6** | **6584** | **6** | **0,58** | **0,586** |  |
|  |  | **2** | **9815** | **6** | **5403** | **6** | **0,55** |  |  |
|  |  | **3** | **9895** | **6** | **6198** | **6** | **0,63** |  |  |
|  | Lab3 | 1 | 2776 | 6 | 1656 | 6 | 0,60 | 0,581 |  |
|  |  | 2 | 7837 | 6 | 4529 | 6 | 0,58 |  |  |
|  |  | 3 | 5000 | 6 | 2844 | 6 | 0,57 |  |  |
| ERM®-BF423d | **Lab1** | **1** | **20326** | **6** | **854** | **6** | **0,04** | **0,043** | 0,04 |
| maize |  | **2** | **14152** | **6** | **630** | **6** | **0,04** |  |  |
|  |  | **3** |  |  |  |  |  |  |  |
|  | Lab2 | 1 | 5381 | 6 | 239 | 6 | 0,04 | 0,045 |  |
|  |  | 2 | 5184 | 6 | 234 | 6 | 0,05 |  |  |
|  |  | 3 | 5787 | 6 | 262 | 6 | 0,05 |  |  |
| 1208-A | **Lab1** | **1** | **35129** | **6** | **21914** | **6** | **0,62** | **0,586** | 0,58 |
| maize |  | **2** | **37774** | **6** | **20039** | **6** | **0,53** |  |  |
|  |  | **3** | **30823** | **6** | **18577** | **6** | **0,60** |  |  |
|  | Lab2 | 1 | 21132 | 6 | 12357 | 6 | 0,58 | 0,569 |  |
|  |  | 2 | 20161 | 6 | 10839 | 6 | 0,54 |  |  |
|  |  | 3 | 21606 | 6 | 12649 | 6 | 0,59 |  |  |
| 0709-A | **Lab1** | **1** | **27983** | **6** | **10616** | **6** | **0,38** | **0,350** | 0,38 |
| maize |  | **2** | **33316** | **6** | **11269** | **6** | **0,34** |  |  |
|  |  | **3** | **39527** | **6** | **13184** | **6** | **0,33** |  |  |
|  | Lab3 | 1 | 4388 | 6 | 1730 | 6 | 0,39 | 0,405 |  |
|  |  | 2 | 3983 | 6 | 1669 | 6 | 0,42 |  |  |
|  |  | 3 | 3895 | 6 | 1566 | 6 | 0,40 |  |  |
| 0512-A | **Lab1** | **1** | **32288** | **6** | **20570** | **6** | **0,64** | **0,602** | 0,58 |
| maize |  | **2** | **38835** | **6** | **22802** | **6** | **0,59** |  |  |
|  |  | **3** | **34289** | **6** | **19988** | **6** | **0,58** |  |  |
|  | Lab2 | 1 | 9661 | 6 | 5696 | 6 | 0,59 | 0,553 |  |
|  |  | 2 | 10108 | 6 | 5594 | 6 | 0,55 |  |  |
|  |  | 3 | 8937 | 6 | 4618 | 6 | 0,52 |  |  |
|  | **Lab3** | **1** | **1929** | **6** | **1046** | **6** | **0,54** | **0,575** |  |
|  |  | **2** | **2631** | **6** | **1551** | **6** | **0,59** |  |  |
|  |  | **3** | **2381** | **6** | **1411** | **6** | **0,59** |  |  |
| ERM®-BF433d | Lab1 | 1 | 13920 | 6 | 481 | 6 | 0,03 | 0,034 | 0,04 |
| maize |  | 2 | 9358 | 6 | 310 | 6 | 0,03 |  |  |
|  |  | 3 | 11700 | 6 | 392 | 6 | 0,03 |  |  |
|  | **Lab2** | **1** | **10980** | **6** | **436** | **6** | **0,04** | **0,039** |  |
|  |  | **2** | **10336** | **6** | **400** | **6** | **0,04** |  |  |
|  |  | **3** | **11471** | **6** | **429** | **6** | **0,04** |  |  |
|  | Lab3 | 1 | 6410 | 6 | 224 | 6 | 0,03 | 0,036 |  |
|  |  | 2 | 6343 | 6 | 242 | 6 | 0,04 |  |  |
|  |  | 3 | 5498 | 6 | 199 | 6 | 0,04 |  |  |
| ERM®-BF420c | **Lab1** | **1** | **8736** | **5** | **404** | **6** | **0,05** | **0,044** | 0,04 |
| maize |  | **2** | **24099** | **6** | **924** | **6** | **0,04** |  |  |
|  |  | **3** | **6948** | **6** | **323** | **6** | **0,05** |  |  |
|  | Lab2 | 1 | 3752 | 6 | 149 | 6 | 0,04 | 0,042 |  |
|  |  | 2 | 3322 | 6 | 152 | 6 | 0,05 |  |  |
|  |  | 3 | 3563 | 6 | 143 | 6 | 0,04 |  |  |
|  | **Lab3** | **1** | **3596** | **6** | **133** | **6** | **0,04** | **0,038** |  |
|  |  | **2** | **4685** | **6** | **179** | **6** | **0,04** |  |  |
|  |  | **3** | **3346** | **6** | **132** | **6** | **0,04** |  |  |
| 0411-D | Lab1 | 1 | 42072 | 6 | 14170 | 6 | 0,34 | 0,347 | 0,36 |
| maize |  | 2 | 39024 | 6 | 13886 | 6 | 0,36 |  |  |
|  |  | 3 | 36513 | 6 | 12725 | 6 | 0,35 |  |  |
|  | **Lab2** | **1** | **6829** | **6** | **2207** | **6** | **0,32** | **0,337** |  |
|  |  | **2** | **6391** | **6** | **2225** | **6** | **0,35** |  |  |
|  |  | **3** | **5993** | **6** | **2037** | **6** | **0,34** |  |  |
|  | Lab3 | 1 | 2977 | 6 | 1212 | 6 | 0,41 | 0,396 |  |
|  |  | 2 | 3525 | 6 | 1311 | 6 | 0,37 |  |  |
|  |  | 3 | 2978 | 6 | 1221 | 6 | 0,41 |  |  |
| ERM®-BF438e | **Lab1** | **1** | **14618** | **6** | **707** | **6** | **0,05** | **0,048** | 0,05 |
| maize |  | **2** | **34090** | **6** | **1523** | **6** | **0,04** |  |  |
|  |  | **3** | **26439** | **6** | **1367** | **6** | **0,05** |  |  |
|  | Lab2 | 1 | 7373 | 6 | 392 | 6 | 0,05 | 0,050 |  |
|  |  | 2 | 5915 | 6 | 292 | 6 | 0,05 |  |  |
|  |  | 3 | 5746 | 6 | 281 | 6 | 0,05 |  |  |
|  | **Lab3** | **1** | **5716** | **6** | **260** | **6** | **0,05** | **0,046** |  |
|  |  | **2** | **5608** | **6** | **259** | **6** | **0,05** |  |  |
|  |  | **3** | **6164** | **6** | **282** | **6** | **0,05** |  |  |
| ERM®-BF411f | Lab1 | 1 | 29639 | 6 | 1051 | 6 | 0,04 | 0,033 | 0,03 |
| maize |  | 2 | 12702 | 6 | 386 | 6 | 0,03 |  |  |
|  |  | 3 | 24963 | 6 | 841 | 6 | 0,03 |  |  |
|  | **Lab3** | **1** | **6278** | **6** | **209** | **6** | **0,03** | **0,035** |  |
|  |  | **2** | **5344** | **6** | **189** | **6** | **0,04** |  |  |
|  |  | **3** | **6184** | **6** | **217** | **6** | **0,04** |  |  |
|  | Lab4 | 1 | 52165 | 3 | 1814 | 6 | 0,03 | 0,034 |  |
|  |  | 2 | 76446 | 4 | 2648 | 6 | 0,03 |  |  |
|  |  | 3 | 67689 | 6 | 2276 | 6 | 0,03 |  |  |
| ERM®-BF416d | **Lab1** | **1** | **22897** | **6** | **1407** | **6** | **0,06** | **0,064** | 0,06 |
| maize |  | **2** | **18947** | **6** | **1252** | **6** | **0,07** |  |  |
|  |  | **3** | **16805** | **6** | **1080** | **6** | **0,06** |  |  |
|  | Lab2 | 1 | 6706 | 6 | 447 | 6 | 0,07 | 0,065 |  |
|  |  | 2 | 6799 | 6 | 445 | 6 | 0,07 |  |  |
|  |  | 3 | 7469 | 6 | 462 | 6 | 0,06 |  |  |
|  | **Lab3** | **1** | **5095** | **6** | **273** | **6** | **0,05** | **0,056** |  |
|  |  | **2** | **3897** | **6** | **218** | **6** | **0,06** |  |  |
|  |  | **3** | **4319** | **6** | **250** | **6** | **0,06** |  |  |
| 0113-A | Lab1 | 1 | 60368 | 6 | 67431 | 6 | 1,12 | 1,061 | 1,09 |
| cotton |  | 2 | 64090 | 6 | 64904 | 6 | 1,01 |  |  |
|  |  | 3 | 59646 | 6 | 62737 | 6 | 1,05 |  |  |
|  | **Lab2** | **1** | **4164** | **5** | **4869** | **6** | **1,17** | **1,167** |  |
|  |  | **2** | **3419** | **6** | **3929** | **6** | **1,15** |  |  |
|  |  | **3** | **3211** | **6** | **3800** | **6** | **1,18** |  |  |
|  | Lab3 | 1 | 16067 | 6 | 16439 | 6 | 1,02 | 1,040 |  |
|  |  | 2 | 12240 | 6 | 13130 | 6 | 1,07 |  |  |
|  |  | 3 | 13999 | 6 | 14350 | 6 | 1,03 |  |  |
| 0804-B | **Lab1** | **1** | **59234** | **6** | **62274** | **6** | **1,05** | **1,045** | 1,05 |
| cotton |  | **2** | **65174** | **6** | **65513** | **6** | **1,01** |  |  |
|  |  | **3** | **59390** | **6** | **64013** | **6** | **1,08** |  |  |
|  | Lab2 | 1 | 7457 | 6 | 8378 | 6 | 1,12 | 1,104 |  |
|  |  | 2 | 7973 | 6 | 8515 | 6 | 1,07 |  |  |
|  |  | 3 | 7355 | 6 | 8248 | 6 | 1,12 |  |  |
|  | **Lab3** | **1** | **12850** | **6** | **12786** | **6** | **0,99** | **1,004** |  |
|  |  | **2** | **15415** | **6** | **15617** | **6** | **1,01** |  |  |
|  |  | **3** | **15634** | **6** | **15681** | **6** | **1,00** |  |  |
| 0804-D | Lab1 | 1 | 43011 | 5 | 43541 | 6 | 1,01 | 1,016 | 0,96 |
| cotton |  | 2 | 44580 | 6 | 44894 | 6 | 1,01 |  |  |
|  |  | 3 | 48147 | 6 | 49457 | 6 | 1,03 |  |  |
|  | **Lab2** | **1** | **8706** | **6** | **7844** | **6** | **0,90** | **0,911** |  |
|  |  | **2** | **6208** | **6** | **5692** | **6** | **0,92** |  |  |
|  |  | **3** | **4400** | **6** | **4022** | **6** | **0,91** |  |  |
|  | Lab3 | 1 | 19292 | 6 | 18440 | 6 | 0,96 | 0,958 |  |
|  |  | 2 | 20965 | 6 | 20323 | 6 | 0,97 |  |  |
|  |  | 3 | 20881 | 6 | 19832 | 6 | 0,95 |  |  |
| 0804-C | **Lab1** | **1** | **51885** | **6** | **56594** | **6** | **1,09** | **1,062** | 0,99 |
| cotton |  | **2** | **50321** | **5** | **52760** | **6** | **1,05** |  |  |
|  |  | **3** | **51495** | **6** | **53835** | **6** | **1,05** |  |  |
|  | Lab2 | 1 | 5830 | 6 | 5499 | 6 | 0,94 | 0,921 |  |
|  |  | 2 | 4934 | 6 | 4552 | 6 | 0,92 |  |  |
|  |  | 3 | 3604 | 6 | 3231 | 6 | 0,90 |  |  |
|  | **Lab3** | **1** | **24701** | **6** | **24133** | **6** | **0,98** | **0,997** |  |
|  |  | **2** | **22021** | **6** | **22136** | **6** | **1,01** |  |  |
|  |  | **3** | **18058** | **6** | **18234** | **6** | **1,01** |  |  |
| ERM®-BF422d | Lab1 | 1 | 34908 | 6 | 3564 | 6 | 0,10 | 0,098 | 0,10 |
| 281-24-236 |  | 2 | 57651 | 6 | 5496 | 6 | 0,10 |  |  |
| cotton |  | 3 | 34306 | 6 | 3296 | 6 | 0,10 |  |  |
|  | **Lab3** | **1** | **12820** | **6** | **1238** | **6** | **0,10** | **0,096** |  |
|  |  | **2** | **12999** | **6** | **1179** | **6** | **0,09** |  |  |
|  |  | **3** | **7981** | **6** | **804** | **6** | **0,10** |  |  |
|  | Lab4 | 1 | 138667 | 6 | 14013 | 6 | 0,10 | 0,102 |  |
|  |  | 2 | 125013 | 5 | 12805 | 5 | 0,10 |  |  |
|  |  | 3 | 90967 | 5 | 9402 | 6 | 0,10 |  |  |
| ERM®-BF422d | **Lab1** | **1** | **34070** | **6** | **3443** | **6** | **0,10** | **0,101** | 0,10 |
| 3006-210-23 |  | **2** | **56850** | **6** | **5693** | **6** | **0,10** |  |  |
| cotton |  | **3** | **35600** | **6** | **3638** | **6** | **0,10** |  |  |
|  | Lab3 | 1 | 12820 | 6 | 1200 | 6 | 0,09 | 0,098 |  |
|  |  | 2 | 12999 | 6 | 1274 | 6 | 0,10 |  |  |
|  |  | 3 | 7981 | 6 | 825 | 6 | 0,10 |  |  |
|  | **Lab4** | **1** | **140681** | **6** | **14679** | **6** | **0,10** | **0,106** |  |
|  |  | **2** | **121304** | **6** | **12905** | **6** | **0,11** |  |  |
|  |  | **3** | **90607** | **6** | **9626** | **6** | **0,11** |  |  |
| ERM®-BF429c | Lab1 | 1 | 54782 | 6 | 6987 | 6 | 0,13 | 0,128 | 0,13 |
| cotton |  | 2 | 54112 | 6 | 7204 | 6 | 0,13 |  |  |
|  |  | 3 | 64772 | 6 | 7934 | 6 | 0,12 |  |  |
|  | **Lab3** | **1** | **11413** | **6** | **1494** | **6** | **0,13** | **0,125** |  |
|  |  | **2** | **11120** | **6** | **1309** | **6** | **0,12** |  |  |
|  |  | **3** | **11367** | **6** | **1435** | **6** | **0,13** |  |  |
|  | Lab4 | 1 | 143730 | 5 | 18625 | 6 | 0,13 | 0,128 |  |
|  |  | 2 | 137863 | 6 | 17548 | 6 | 0,13 |  |  |
|  |  | 3 | 152381 | 6 | 19320 | 6 | 0,13 |  |  |
| 0906-D | **Lab1** | **1** | **43332** | **6** | **42501** | **6** | **0,98** | **0,994** | 1,02 |
| cotton |  | **2** | **43360** | **6** | **43407** | **5** | **1,00** |  |  |
|  |  | **3** | **46012** | **6** | **46073** | **6** | **1,00** |  |  |
|  | Lab2 | 1 | 3390 | 6 | 3627 | 6 | 1,07 | 1,072 |  |
|  |  | 2 | 2334 | 5 | 2524 | 6 | 1,08 |  |  |
|  |  | 3 | 5797 | 6 | 6176 | 6 | 1,07 |  |  |
|  | **Lab3** | **1** | **8559** | **5** | **8678** | **6** | **1,01** | **1,000** |  |
|  |  | **2** | **7677** | **6** | **7533** | **6** | **0,98** |  |  |
|  |  | **3** | **7015** | **6** | **7042** | **6** | **1,00** |  |  |
| ERM®-BF432d | Lab1 | 1 | 51106 | 6 | 6393 | 6 | 0,13 | 0,119 | 0,12 |
| soybean |  | 2 | 59284 | 6 | 6902 | 6 | 0,12 |  |  |
|  |  | 3 | 59192 | 6 | 6858 | 6 | 0,12 |  |  |
|  | **Lab2** | **1** | **29136** | **6** | **3009** | **6** | **0,10** | **0,115** |  |
|  |  | **2** | **27718** | **6** | **3042** | **6** | **0,11** |  |  |
|  |  | **3** | **24052** | **6** | **3151** | **6** | **0,13** |  |  |
|  | Lab3 | 1 | 14590 | 6 | 1739 | 6 | 0,12 | 0,118 |  |
|  |  | 2 | 14283 | 6 | 1620 | 6 | 0,11 |  |  |
|  |  | 3 | 12146 | 6 | 1485 | 5 | 0,12 |  |  |
| ERM®-BF436e | **Lab1** | **1** | **62245** | **6** | **6688** | **6** | **0,11** | **0,108** | 0,10 |
| soybean |  | **2** | **63645** | **6** | **6684** | **6** | **0,11** |  |  |
|  |  | **3** | **55020** | **6** | **6112** | **6** | **0,11** |  |  |
|  | Lab2 | 1 | 30022 | 6 | 2502 | 6 | 0,08 | 0,087 |  |
|  |  | 2 | 32681 | 6 | 2910 | 6 | 0,09 |  |  |
|  |  | 3 | 27885 | 6 | 2468 | 6 | 0,09 |  |  |
|  |  |  |  |  |  |  |  |  |  |
|  | **Lab3** | **1** | **12356** | **6** | **1253** | **6** | **0,10** | **0,100** |  |
|  |  | **2** | **12456** | **6** | **1235** | **6** | **0,10** |  |  |
|  |  | **3** | **13640** | **6** | **1353** | **6** | **0,10** |  |  |
|  | Lab4 | 1 | 67900 | 6 | 6871 | 6 | 0,10 | 0,100 |  |
|  |  | 2 | 125649 | 6 | 12552 | 6 | 0,10 |  |  |
|  |  | 3 | 94918 | 6 | 9277 | 6 | 0,10 |  |  |
| ERM®-BF437e | **Lab1** | **1** | **55925** | **6** | **5186** | **6** | **0,09** | **0,089** | 0,09 |
| soybean |  | **2** | **55376** | **6** | **4964** | **6** | **0,09** |  |  |
|  |  | **3** | **50729** | **6** | **4250** | **6** | **0,08** |  |  |
|  | Lab2 | 1 | 26936 | 6 | 2219 | 6 | 0,08 | 0,083 |  |
|  |  | 2 | 27138 | 6 | 2353 | 6 | 0,09 |  |  |
|  |  | 3 | 26651 | 6 | 2140 | 6 | 0,08 |  |  |
|  | **Lab3** | **1** | **10682** | **6** | **846** | **6** | **0,08** | **0,084** |  |
|  |  | **2** | **12501** | **6** | **1046** | **6** | **0,08** |  |  |
|  |  | **3** | **11728** | **6** | **1034** | **6** | **0,09** |  |  |
| 0906-B | **Lab1** | **1** | **51794** | **6** | **48245** | **6** | **0,93** | **0,939** | 0,98 |
| soybean |  | **2** | **44403** | **6** | **42172** | **6** | **0,95** |  |  |
|  |  | **3** | **44093** | **6** | **41279** | **6** | **0,94** |  |  |
|  | Lab2 | 1 | 83890 | 6 | 87870 | 6 | 1,05 | 1,028 |  |
|  |  | 2 | 82542 | 6 | 84916 | 6 | 1,03 |  |  |
|  |  | 3 | 85535 | 6 | 86231 | 6 | 1,01 |  |  |
|  | **Lab3** | **1** | **8117** | **6** | **7913** | **6** | **0,97** | **0,972** |  |
|  |  | **2** | **5753** | **6** | **5611** | **6** | **0,98** |  |  |
|  |  | **3** | **8667** | **6** | **8377** | **6** | **0,97** |  |  |
| ERM®-BF410ep | Lab1 | 1 | 69151 | 6 | 6632 | 6 | 0,10 | 0,091 | 0,08 |
| soybean |  | 2 | 66781 | 6 | 6169 | 5 | 0,09 |  |  |
|  |  | 3 | 67597 | 5 | 5636 | 6 | 0,08 |  |  |
|  | **Lab2** | **1** | **37727** | **6** | **2587** | **6** | **0,07** | **0,065** |  |
|  |  | **2** | **36230** | **6** | **2340** | **6** | **0,06** |  |  |
|  |  | **3** | **37922** | **6** | **2328** | **6** | **0,06** |  |  |
|  | Lab3 | 1 | 24080 | 6 | 1942 | 6 | 0,08 | 0,080 |  |
|  |  | 2 | 21343 | 6 | 1658 | 6 | 0,08 |  |  |
|  |  | 3 | 17091 | 6 | 1389 | 6 | 0,08 |  |  |
|  | **Lab4** | **1** | **101961** | **6** | **8426** | **6** | **0,08** | **0,083** |  |
|  |  | **2** | **68429** | **4** | **5346** | **6** | **0,08** |  |  |
|  |  | **3** | **154751** | **3** | **13488** | **6** | **0,09** |  |  |
| 0809-A | Lab1 | 1 | 53487 | 6 | 50439 | 6 | 0,94 | 0,917 | 0,95 |
| soybean |  | 2 | 48896 | 6 | 44914 | 6 | 0,92 |  |  |
|  |  | 3 | 44824 | 6 | 39894 | 6 | 0,89 |  |  |
|  | **Lab2** | **1** | **74989** | **6** | **70342** | **6** | **0,94** | **0,961** |  |
|  |  | **2** | **64328** | **6** | **61929** | **6** | **0,96** |  |  |
|  |  | **3** | **62585** | **6** | **61537** | **6** | **0,98** |  |  |
|  | Lab3 | 1 | 7828 | 6 | 7786 | 6 | 0,99 | 0,958 |  |
|  |  | 2 | 8489 | 6 | 7956 | 6 | 0,94 |  |  |
|  |  | 3 | 7511 | 6 | 7087 | 6 | 0,94 |  |  |
| ERM®-BF425d | **Lab1** | **1** | **48088** | **6** | **4958** | **5** | **0,10** | **0,105** | 0,10 |
| soybean |  | **2** | **53293** | **6** | **6046** | **6** | **0,11** |  |  |
|  |  | **3** | **53874** | **6** | **5279** | **6** | **0,10** |  |  |
|  | Lab2 | 1 | 35247 | 6 | 3134 | 6 | 0,09 | 0,092 |  |
|  |  | 2 | 31488 | 6 | 2946 | 6 | 0,09 |  |  |
|  |  | 3 | 30240 | 6 | 2854 | 6 | 0,09 |  |  |
|  | **Lab3** | **1** | **15174** | **5** | **1517** | **6** | **0,10** | **0,097** |  |
|  |  | **2** | **12581** | **6** | **1228** | **6** | **0,10** |  |  |
|  |  | **3** | **14307** | **6** | **1355** | **6** | **0,09** |  |  |
| 0210-A | **Lab1** | **1** | **39171** | **6** | **36969** | **6** | **0,94** | **1,004** | 0,96 |
| soybean |  | **2** | **37084** | **6** | **37773** | **6** | **1,02** |  |  |
|  |  | **3** | **37129** | **6** | **38937** | **6** | **1,05** |  |  |
|  | Lab2 | 1 | 30709 | 6 | 27942 | 6 | 0,91 | 0,925 |  |
|  |  | 2 | 11886 | 6 | 11195 | 6 | 0,94 |  |  |
|  |  | 3 | 43252 | 6 | 39971 | 6 | 0,92 |  |  |
|  | **Lab3** | **1** | **9453** | **6** | **8859** | **6** | **0,94** | **0,959** |  |
|  |  | **2** | **7548** | **6** | **7425** | **6** | **0,98** |  |  |
|  |  | **3** | **7399** | **6** | **7071** | **6** | **0,96** |  |  |
| 0311-A | Lab1 | 1 | 38165 | 6 | 41702 | 6 | 1,09 | 1,069 | 1,00 |
| soybean |  | 2 | 35432 | 6 | 37286 | 6 | 1,05 |  |  |
|  |  | 3 | 37201 | 6 | 39472 | 6 | 1,06 |  |  |
|  | **Lab2** | **1** | **62845** | **6** | **62955** | **6** | **1,00** | **0,951** |  |
|  |  | **2** | **65453** | **6** | **58899** | **6** | **0,90** |  |  |
|  |  | **3** | **63729** | **6** | **60668** | **6** | **0,95** |  |  |
|  | Lab3 | 1 | 6701 | 6 | 6176 | 6 | 0,92 | 0,966 |  |
|  |  | 2 | 8352 | 6 | 8174 | 6 | 0,98 |  |  |
|  |  | 3 | 10820 | 6 | 10785 | 6 | 1,00 |  |  |
| 0809-B | **Lab1** | **1** | **38596** | **6** | **40730** | **6** | **1,06** | **1,033** | 0,99 |
| soybean |  | **2** | **33282** | **6** | **33658** | **6** | **1,01** |  |  |
|  |  | **3** | **26933** | **6** | **27783** | **6** | **1,03** |  |  |
|  | Lab2 | 1 | 82540 | 6 | 81008 | 6 | 0,98 | 0,962 |  |
|  |  | 2 | 84032 | 6 | 83326 | 6 | 0,99 |  |  |
|  |  | 3 | 72901 | 6 | 66489 | 6 | 0,91 |  |  |
|  | **Lab3** | **1** | **8079** | **6** | **7676** | **6** | **0,95** | **0,972** |  |
|  |  | **2** | **7468** | **6** | **7436** | **6** | **1,00** |  |  |
|  |  | **3** | **7074** | **6** | **6857** | **6** | **0,97** |  |  |
| ERM®-BF426d | Lab1 | 1 | 59208 | 6 | 6026 | 6 | 0,10 | 0,100 | 0,09 |
| soybean |  | 2 | 43982 | 6 | 4512 | 6 | 0,10 |  |  |
|  |  | 3 | 53393 | 6 | 5078 | 6 | 0,10 |  |  |
|  |  |  |  |  |  |  |  |  |  |
|  | **Lab2** | **1** | **26713** | **6** | **2187** | **6** | **0,08** | **0,082** |  |
|  |  | **2** | **29782** | **6** | **2444** | **6** | **0,08** |  |  |
|  |  | **3** | **27440** | **6** | **2283** | **6** | **0,08** |  |  |
|  | Lab3 | 1 | 14061 | 6 | 1332 | 6 | 0,09 | 0,094 |  |
|  |  | 2 | 13572 | 6 | 1226 | 6 | 0,09 |  |  |
|  |  | 3 | 13966 | 6 | 1350 | 6 | 0,10 |  |  |
|  | **Lab4** | **1** | **103280** | **6** | **9883** | **6** | **0,10** | **0,095** |  |
|  |  | **2** | **111888** | **6** | **10686** | **6** | **0,10** |  |  |
|  |  | **3** | **107761** | **6** | **10145** | **6** | **0,09** |  |  |
| 0911-D | Lab1 | 1 | 40192 | 6 | 45772 | 6 | 1,14 | 1,082 | 1,01 |
| soybean |  | 2 | 48013 | 6 | 47038 | 6 | 0,98 |  |  |
|  |  | 3 | 33255 | 6 | 37477 | 6 | 1,13 |  |  |
|  | **Lab2** | **1** | **12978** | **6** | **13534** | **6** | **1,04** | **0,982** |  |
|  |  | **2** | **26231** | **6** | **25447** | **6** | **0,97** |  |  |
|  |  | **3** | **22477** | **6** | **20990** | **6** | **0,93** |  |  |
|  | Lab3 | 1 | 5274 | 6 | 5032 | 6 | 0,95 | 0,966 |  |
|  |  | 2 | 4317 | 6 | 4197 | 6 | 0,97 |  |  |
|  |  | 3 | 5453 | 6 | 5294 | 6 | 0,97 |  |  |
| ERM®-BF434e | Lab1 | 1 | 76756 | 6 | 8331 | 6 | 0,11 | 0,109 | 0,09 |
| *oilseed rape* |  | 2 | 87540 | 6 | 9404 | 6 | 0,11 |  |  |
|  |  | 3 | 90054 | 5 | 9866 | 6 | 0,11 |  |  |
|  | **Lab2** | **1** | **19681** | **6** | **1735** | **6** | **0,09** | **0,081** |  |
|  |  | **2** | **18188** | **6** | **1381** | **6** | **0,08** |  |  |
|  |  | **3** | **18988** | **6** | **1517** | **6** | **0,08** |  |  |
|  | Lab3 | 1 | 9806 | 6 | 863 | 6 | 0,09 | 0,085 |  |
|  |  | 2 | 8283 | 6 | 703 | 6 | 0,08 |  |  |
|  |  | 3 | 10499 | 5 | 869 | 6 | 0,08 |  |  |
|  | Lab4 | 1 | 134500 | 6 | 13859 | 6 | 0,10 | 0,104 |  |
|  |  | 2 | 127136 | 6 | 13332 | 6 | 0,10 |  |  |
|  |  | 3 | 122054 | 6 | 12712 | 6 | 0,10 |  |  |
| 0304-B2 | **Lab1** | **1** | **67182** | **6** | **56857** | **6** | **0,85** | **0,872** | 0,92 |
| *oilseed rape* |  | **2** | **72608** | **6** | **61340** | **6** | **0,84** |  |  |
|  |  | **3** | **65657** | **6** | **60821** | **6** | **0,93** |  |  |
|  | Lab2 | 1 | 69650 | 6 | 60904 | 6 | 0,87 | 0,897 |  |
|  |  | 2 | 73975 | 6 | 64672 | 6 | 0,87 |  |  |
|  |  | 3 | 72404 | 6 | 68274 | 6 | 0,94 |  |  |
|  | Lab3 | 1 | 9023 | 6 | 8997 | 6 | 1,00 | 0,991 |  |
|  |  | 2 | 8149 | 6 | 7825 | 6 | 0,96 |  |  |
|  |  | 3 | 8762 | 6 | 8903 | 6 | 1,02 |  |  |
| 1011-A | **Lab1** | **1** | **74259** | **6** | **72786** | **5** | **0,98** | **0,984** | 0,96 |
| *oilseed rape* |  | **2** | **41992** | **6** | **42914** | **6** | **1,02** |  |  |
|  |  | **3** | **63501** | **6** | **60318** | **6** | **0,95** |  |  |
|  |  |  |  |  |  |  |  |  |  |
|  | Lab2 | 1 | 35326 | 6 | 35509 | 6 | 1,01 | 0,959 |  |
|  |  | 2 | 57880 | 6 | 53877 | 6 | 0,93 |  |  |
|  |  | 3 | 45361 | 6 | 42743 | 6 | 0,94 |  |  |
|  | Lab3 | 1 | 6557 | 6 | 6371 | 6 | 0,97 | 0,951 |  |
|  |  | 2 | 7976 | 6 | 7398 | 6 | 0,93 |  |  |
|  |  | 3 | 6900 | 6 | 6585 | 6 | 0,95 |  |  |
| ERM®-BF419b | **Lab1** | **1** | **19618** | **6** | **9125** | **6** | **0,47** | **0,471** | 0,48 |
| Sugar beet |  | **2** | **43632** | **6** | **20229** | **6** | **0,46** |  |  |
|  |  | **3** | **17009** | **5** | **8220** | **6** | **0,48** |  |  |
|  | Lab2 | 1 | 16174 | 6 | 7942 | 6 | 0,49 | 0,449 |  |
|  |  | 2 | 11886 | 6 | 5146 | 6 | 0,43 |  |  |
|  |  | 3 | 12651 | 6 | 5358 | 6 | 0,42 |  |  |
|  | Lab3 | 1 | 6770 | 6 | 3523 | 6 | 0,52 | 0,506 |  |
|  |  | 2 | 6380 | 6 | 3054 | 6 | 0,48 |  |  |
|  |  | 3 | 7836 | 6 | 4055 | 6 | 0,52 |  |  |
| ERM®-BF428c | **Lab1** | **1** | **49461** | **6** | **5530** | **6** | **0,11** | **0,114** | **Not calculated** |
| cotton |  | **2** | **40757** | **6** | **4721** | **6** | **0,12** |  |  |
|  |  | **3** | **44845** | **6** | **5163** | **6** | **0,12** |  |  |
|  | Lab2 | 1 | 12564 | 6 | 974 | 6 | 0,08 | 0,073 |  |
|  |  | 2 | 15040 | 6 | 1068 | 6 | 0,07 |  |  |
|  |  | 3 | 15790 | 6 | 1113 | 6 | 0,07 |  |  |
|  | **Lab3** | **1** | **10258** | **6** | **1024** | **6** | **0,10** | **0,098** |  |
|  |  | **2** | **7709** | **6** | **734** | **6** | **0,10** |  |  |
|  |  | **3** | **9124** | **6** | **890** | **6** | **0,10** |  |  |
|  | Lab4 | 1 | 96899 | 6 | 10845 | 6 | 0,11 | 0,103 |  |
|  |  | 2 | 141573 | 4 | 14117 | 6 | 0,10 |  |  |
|  |  | 3 | 131210 | 6 | 12914 | 5 | 0,10 |  |  |

**Table S1.2:** Detailed data providing the average copy number concentrations (cp/µL) per laboratory for both endogen and taxon-specific assays as well as the calculated endogen/taxon-specific ratio, mean ratio per lab and overall mean ratio used for the determination of the conversion factors in CRM provided as genomic DNA solutions.

| **CRM** | **Lab** | **transgen** | **Replicates transgen** | **Taxon** | **Replicates Taxon** | **ratio** | **overall mean ratio** |
| --- | --- | --- | --- | --- | --- | --- | --- |
|  |  | [cp/μl] |  | [cp/μl] |  |  |  |
| 0306-I8 | Lab1 | 74645 | 5 | 56085 | 6 | 0,75 | 0,82 |
| rice | **Lab2** | **38466** | **6** | **34654** | **6** | **0,90** |  |
|  | Lab3 | 59299 | 6 | 48233 | 6 | 0,81 |  |
|  | **Lab4** | **232401** | **5** | **192730** | **5** | **0,83** |  |
| 0306-E2 | Lab1 | 13998 | 6 | 14076 | 6 | 1,01 | 1,00 |
| cotton | **Lab3** | **9434** | **4** | **9377** | **6** | **0,99** |  |
|  | Lab4 | 50179 | 5 | 50584 | 6 | 1,01 |  |
| 1108-A5 | **Lab1** | **26237** | **6** | **28593** | **6** | **1,09** | 1,11 |
| cotton | Lab2 | 14376 | 6 | 16758 | 6 | 1,17 |  |
|  | **Lab3** | **18026** | **6** | **19431** | **6** | **1,08** |  |
| 0707-B10 | Lab1 | 150614 | 6 | 147807 | 6 | 0,98 | 0,97 |
| soybean | **Lab3** | **186358** | **6** | **185137** | **6** | **0,99** |  |
|  | Lab4 | 586403 | 5 | 555233 | 6 | 0,95 |  |
| 0610-A4 | **Lab1** | **31647** | **5** | **30758** | **5** | **0,97** | 1,03 |
| soybean | Lab2 | 15808 | 6 | 17570 | 6 | 1,11 |  |
|  | **Lab3** | **118013** | **6** | **118614** | **6** | **1,01** |  |
| 0306-F6 | Lab1 | 163150 | 6 | 85373 | 6 | 0,52 | 0,50 |
| canola | **Lab2** | **40902** | **6** | **19255** | **6** | **0,47** |  |
|  | Lab3 | 24351 | 6 | 12561 | 6 | 0,52 |  |
| 0306-G5 | Lab1 | 135056 | 6 | 134780 | 6 | 1,00 | 1,01 |
| canola | **Lab2** | **9888** | **6** | **10686** | **6** | **1,08** |  |
|  | Lab3 | 53847 | 6 | 51723 | 6 | 0,96 |  |
| 0208-A5 | **Lab1** | **150601** | **6** | **147025** | **6** | **0,98** | 0,95 |
| canola | Lab2 | 13543 | 6 | 12653 | 6 | 0,93 |  |
|  | Lab3 | 34582 | 6 | 32023 | 6 | 0,93 |  |
| 0306-H9 | **Lab1** | **13062** | **4** | **7394** | **6** | **0,57** | Not calculated |
| maize | Lab2 | 6547 | 6 | 6099 | 6 | 0,93 |  |
|  | **Lab3** | **9481** | **6** | **7062** | **6** | **0,74** |  |
|  | Lab4 | 38008 | 6 | 28973 | 6 | 0,76 |  |
| 0707-C6 | Lab1 | **59321** | **6** | **61181** | **6** | **1,03** | 1,01 |
| soybean | Lab2 | **63707** | **6** | **60043** | **6** | **0,94** |  |
|  | Lab3 | **54062** | **6** | **57032** | **6** | **1,05** |  |

**Table S****1.3:** Oligonucleotides used as targets for the ddPCR taxon-specific methods. A fluorescent marker, quencher or oligo sequence coloured in red indicates a difference with the qPCR method published in the EURL GMFF method database.

| **Target gene (Taxon)** | **Oligonucleotide^[[1]](#footnote-1)^** | **Concentration2 (nM)** | **Nucleotide sequence (5'->3')** | **Amplicon Size (bp)** | **Corresponding qPCR GMFF method** |
| --- | --- | --- | --- | --- | --- |
| phospholipase D - PLD  (*Oryza sativa*) | Forward  Reverse  Probe (1, 3, 4)  Probe (2) | 200 (1, 2, 3) - 400 (4)  200 (1, 2, 3) - 400 (4)  200  200 | TGGTGAGCGTTTTGCAGTCT  CTGATCCACTAGCAGGAGGTCC  6FAM-TGTTGTGCTGCCAATGTGGCCTG-TAMRA  VIC-TGTTGTGCTGCCAATGTGGCCTG-TAMRA | 68 | QT-TAX-OS-017 |
| high mobility group protein A - hmgA  (*Zea mays*) | Forward  Reverse  Probe (1, 4)  Probe (2)  Probe (3) | 300 (2) - 400 (1, 4) - 900 (3)  300 (2) - 400 (1, 4) - 900 (3)  150  160  250 | TTGGACTAGAAATCTCGTGCTGA  GCTACATAGGGAGCCTTGTCCT  6FAM-CAATCCACACAAACGCACGCGTA-TAMRA  6FAM-CAATCCACACAAACGCACGCGTA-BHQ1^[[2]](#footnote-2)^  HEX-CAATCCACACAAACGCACGCGTA-BHQ1 | 79 | QT-TAX-ZM-002 |
| alcohol dehydrogenase - ZmAdh1^[[3]](#footnote-3)^ (*Zea mays*) | Forward (3)  Reverse (3)  Probe (3) | 900  900  250 | CGTCGTTTCCCATCTCTTCCTCC  CGTCGTTTCCCATCTCTTCCTCC  HEX-AATCAGGGCTCATTTTCTCGCTCCTCA-BHQ1 | 135 | QT-TAX-ZM-003 |
| alcohol dehydrogenase - AdhC (*Gossypium hirsutum*) | Forward  Reverse  Probe (1, 3, 4)  Probe (2) | 200 (1, ,2 ,4)-900 (3)  200 (1, 2, 4)– 900 (3)  200  200 | CACATGACTTAGCCCATCTTTGC  CCCACCCTTTTTTGGTTTAGC  6FAM-TGCAGGTTTTGGTGCCACTGTGAATG-TAMRA  6FAM-TGCAGGTTTTGGTGCCACTGTGAATG-BHQ1 | 73 | QT-TAX-GH-018 |
| lectin - Le1  (*Glycine max*) | Forward (1, 2, 4)  Forward (3)  Reverse (1, 2, 4)  Reverse (3)  Probe (1, 4)  Probe (2)  Probe (3) | 650  900  650  900  180  180  250 | CCAGCTTCGCCGCTTCCTTC  CACCTTTCTCGCACCAATTGACA  GAAGGCAAGCCCATCTGCAAGCC  TCAAACTCAACAGCGACGAC  6FAM-CTTCACCTTCTATGCCCCTGACAC-TAMRA  6FAM-CTTCACCTTCTATGCCCCTGACAC-BHQ1  6FAM-CCACAAACACATGCAGGTTATCTTGG-TAMRA | 74 (1, 2, 4)  105 (3) | QT-TAX-GM-002 QT-TAX-GM-009 |
| Acyl-[acyl-carrier-protein] hydrolase - FatA(A)  (*Brassica rapa*) | Forward  Reverse  Probe (1,2, 4)  Probe (3) | 300 (1, 2, 4) – 900 (3)  900  150  250 | ACAGATGAAGTTCGGGACGAGTAC CAGGTTGAGATCCACATGCTTAAATAT  6FAM-AAGAAGAATCATCATGCTTC-MGBNFQ^[[4]](#footnote-4)^  HEX-AAGAAGAATCATCATGCTTC-MGBEQ^[[5]](#footnote-5)^ | 126 | QT-TAX-BN-001 |
| Glutamate dehydrogenase - GluD (*Beta vulgaris*) | Forward  Reverse  Probe (1,2)  Probe (3) | 150 (1,2) – 900 (3)  150 (1,2) – 900 (3)  100  250 | GACCTCCATATTACTGAAAGGAAG GAGTAATTGCTCCATCCTGTTCA  6FAM-CTACGAAGTTTAAAGTATGTGCCGCTC-TAMRA  HEX-CTACGAAGTTTAAAGTATGTGCCGCTC-BHQ1 | 118 | QT-TAX-BV-003 |

**Table S1.4:** Oligonucleotides used as targets for the ddPCR GM maize methods. A fluorescent marker, quencher or oligo sequence coloured in red indicates a difference with the qPCR method published in the EURL GMFF method database.

| **GM maize event** | **Oligonucleotide^[[6]](#footnote-6)^** | **Concentration2 (nM)** | **Nucleotide sequence (5'->3')** | **Amplicon Size (bp)** | **Corresponding qPCR GMFF method** |
| --- | --- | --- | --- | --- | --- |
| Bt11 | Forward (1, 3, 4)  Forward (2)  Reverse (1, 3, 4)  Reverse (2)  Probe (1, 4)  Probe (2)  Probe (3) | 200 (1) – 900 (3) – 400 (4)  600  200 (1) – 900 (3) – 400 (4)  600  150 (1) – 200 (4)  150  250 | TGTGTGGCCATTTATCATCGA  GCGGCTTATCTGTCTCAGGG  CGCTCAGTGGAACGAAAACTC  CAACTGGTCTCCTCTCCGGA  6FAM-TTCCATGACCAAAATCCCTTAACGTGAGT-TAMRA  6FAM-CGTGTTCCCTCGGATCTCGACATGT–TAMRA  6FAM-TTCCATGACCAAAATCCCTTAACGTGAGT-BHQ1 | 68 (1, 3, 4)  75 (2) | QT-EVE-ZM-015 J. AOAC Int. 85, 646 |
| DAS59122 | Forward  Reverse  Probe (1, 2, 4)  Probe (3) | 250 (1, 2, 4) – 900 (3)  250 (1, 2, 4) – 900 (3)  200  250 | GGGATAAGCAAGTAAAAGCGCTC  CCTTAATTCTCCGCTCATGATCAG  6FAM-TTTAAACTGAAGGCGGGAAACGACAA–TAMRA  6FAM-TTTAAACTGAAGGCGGGAAACGACAA–BHQ1 | 86 | QT-EVE-ZM-012 |
| 1507 | Forward (1, 4)  Forward (2, 3)  Reverse (1, 4)  Reverse (2, 3)  Probe (1, 2, 4)  Probe (3) | 300  900  300  900  150 (1, 4) – 200 (2)  250 | TAGTCTTCGGCCAGAATGG  TAGTCTTCGGCCAGAATGG  CTTTGCCAAGATCAAGCG  CTTTGCCAAGATCAAGCG  6FAM-TAACTCAAGGCCCTCACTCCG–TAMRA  6FAM-TAACTCAAGGCCCTCACTCCG–BHQ1 | 58 | QT-EVE-ZM-010 |
| GA21 | Forward (1, 3)  Forward (2, 4)  Reverse (1, 3)  Reverse (2, 4)  Probe (1)  Probe (2, 4)  Probe (3) | 900  900  900  900  300  200 (2) – 300 (4)  250 | CTTATCGTTATGCTATTTGCAACTTTAGA  CGTTATGCTATTTGCAACTTTAGAACA  TGGCTCGCGATCCTCCT  GCGATCCTCCTCGCGTT  6FAM-CATATACTAACTCATATCTCTTTCTCAACAGCAGGTGGGT-TAMRA  6FAM-TTTCTCAACAGCAGGTGGGTCCGGGT-TAMRA  6FAM-CATATACTAACTCATATCTCTTTCTCAACAGCAGGTGGGT-BHQ1 | 112 (1, 3) 101 (2,4) | QT-EVE-ZM-007 QT-EVE-ZM-014 |
| NK603 | Forward (1)  Forward (2, 3)  Reverse (1)  Reverse (2, 3)  Probe (1, 2)  Probe (3) | 150  900  150  900  50 (1) – 200 (2)  250 | ATGAATGACCTCGAGTAAGCTTGTTAA  ATGAATGACCTCGAGTAAGCTTGTTAA  AAGAGATAACAGGATCCACTCAAACACT  AAGAGATAACAGGATCCACTCAAACACT  6FAM-TGGTACCACGCGACACACTTCCACTC–TAMRA  6FAM-TGGTACCACGCGACACACTTCCACTC–BHQ1 | 108 | QT-EVE-ZM-008 |
| MON88017 | Forward  Reverse  Probe (1, 2, 4)  Probe (3) | 150 (1, 4) – 450 (2) – 900 (3)  150 (1, 4) – 450 (2) – 900 (3)  50 (1, 4) – 150 (2)  250 | GAGCAGGACCTGCAGAAGCT  TCCGGAGTTGACCATCCA  6FAM-TCCCGCCTTCAGTTTAAACAGAGTCGGGT-TAMRA  6FAM-TCCCGCCTTCAGTTTAAACAGAGTCGGGT-BHQ1 | 95 | QT-EVE-ZM-016 |
| MON89034 | Forward  Reverse  Probe (1, 2)  Probe (3) | 400 (1) – 450 (2) – 900 (4)  400 (1) – 450 (2) – 900 (4)  150 (1) – 100 (2)  250 | TTCTCCATATTGACCATCATACTCATT  CGGTATCTATAATACCGTGGTTTTTAAA  6FAM-ATCCCCGGAAATTATGTT-MGBNFQ  6FAM-ATCCCCGGAAATTATGTT-MGBEQ | 77 | QT-EVE-ZM-018 |
| MIR604 | Forward  Reverse  Probe (1, 2)  Probe (3) | 600 (1,2) – 900 (3)  300 (1, 2) – 900 (3)  100 (1) – 200 (2)  250 | GCGCACGCAATTCAACAG  GGTCATAACGTGACTCCCTTAATTCT  6FAM-AGGCGGGAAACGACAATCTGATCATG-TAMRA  6FAM-AGGCGGGAAACGACAATCTGATCATG-BHQ1 | 76 | QT-EVE-ZM-013 |
| MIR162 | Forward  Reverse  Probe (1)  Probe (2)  Probe (3) | 900 (1, 3) – 300 (2)  900 (1, 3) – 300 (2)  300  150  250 | GCGCGGTGTCATCTATGTTACTAG  TGCCTTATCTGTTGCCTTCAGA  6FAM-TCTAGACAATTCAGTACATTAAAAACGTCCGCCA-TAMRA  HEX-TCTAGACAATTCAGTACATTAAAAACGTCCGCCA-BHQ1  6FAM-TCTAGACAATTCAGTACATTAAAAACGTCCGCCA-BHQ1 | 92 | QT-EVE-ZM-022 |
| MON87460 | Forward  Reverse  Probe (1)  Probe (2)  Probe (3) | 900 (1, 3) – 600 (2)  900 (1, 3) – 600 (2)  300  250  250 | CACGTTGAAGGAAAATGGATTG  TCGCGATCCTCCTCAAAGAC  6FAM-AGGGAGTATGTAGATAAATTTTCAAAGCGTTAGACGGC-TAMRA  HEX-AGGGAGTATGTAGATAAATTTTCAAAGCGTTAGACGGC-BHQ1  6FAM-AGGGAGTATGTAGATAAATTTTCAAAGCGTTAGACGGC-BHQ1 | 82 | QT-EVE-ZM-005 |
| MON87427 | Forward  Reverse  Probe (1)  Probe (2)  Probe (3) | 900 (1, 3) – 450 (2)  900 (1, 3) – 450 (2)  300  200  250 | ACGGAAACGGTCGGGTCAAATG  CCATGTAGATTTCCCGGTTTTCTC  6FAM-TCGGGACAATATGGAGAAAAAGAAAGAG-TAMRA  HEX-TCGGGACAATATGGAGAAAAAGAAAGAG-BHQ1  6FAM-TCGGGACAATATGGAGAAAAAGAAAGAG-BHQ1 | 95 | QT-EVE-ZM-003 |
| DAS-4Ø278-9 | Forward  Reverse  Probe (1)  Probe (2)  Probe (3) | 350 (1, 2) – 900 (3)  350 (1, 2) – 900 (3)  150  150  250 | CACGAACCATTGAGTTACAATC  TGGTTCATTGTATTCTGGCTTTG  6FAM-CGTAGCTAACCTTCATTGTATTCCG-BHQ1  HEX-CGTAGCTAACCTTCATTGTATTCCG-BHQ1  6FAM-CGTAGCTAACCTTCATTGTATTCCG-TAMRA | 98 | QT-EVE-ZM-004 |
| 3272 | Forward  Reverse  Probe (1, 2)  Probe (3) | 50 (1) – 900 (2, 3)  900  200  250 | TCATCAGACCAGATTCTCTTTTATGG  CGTTTCCCGCCTTCAGTTTA  6FAM-ACTGCTGACGCGGCCAAACACTG-TAMRA  6FAM-ACTGCTGACGCGGCCAAACACTG-BHQ1 | 95 | QT-EVE-ZM-019 |
| 5307 | Forward  Reverse  Probe (1, 2)  Probe (3) | 350 (1, 2) – 900 (3)  350 (1, 2) – 900 (3)  125  250 | CATGGCCGTATCCGCAATGTG  TGCACCCTTTGCCAGTGG  6FAM-ACCACAATATACCCTCTTCCCTGGGCCAG-TAMRA  6FAM-ACCACAATATACCCTCTTCCCTGGGCCAG-BHQ1 | 107 | QT-EVE-ZM-002 |
| VCO01981 | Forward  Reverse  Probe (1)  Probe (2)  Probe (3) | 350 (1) – 300 (2) – 900 (3)  350 (1) – 300 (2) – 900 (3)  150  150  250 | CACGAACCATTGAGTTACAATC  TGGTTCATTGTATTCTGGCTTTG  VIC-CAGTACTCAAACACTGATAG-MGB  6FAM-CAGTACTCAAACACTGATAG-MGB  6FAM-CAGTACTCAAACACTGATAG-MGBEQ | 85 | QT-EVE-ZM-001 |
| Bt176 | Forward (1, 3, 4)  Forward (2)  Reverse (1, 3, 4)  Reverse (2)  Probe (1, 4)  Probe (3)  Probe (2) | 300 (1) – 900 (3) – 400 (4)  900  300 (1) – 900 (3) – 400 (4)  900  200  250  200 | GGCCGTGAACGAGCTGTT  CTCGCTTCCGTGCTTAGCTT  GGGAAGAAGCCTACATGTTTTCTAA  ATGCACTCGTTGATGTTGGG  6FAM-AGCAACCAGATCGGCCGACACC-TAMRA  6FAM-AGCAACCAGATCGGCCGACACC-BHQ1  6FAM-CCGCCGCGGGATCCAACAA-TAMRA | 82 (1, 3, 4)  72 (2) | "J. AOAC Int. 85, 646  QT-EVE-ZM-023" |
| MON863 | Forward  Reverse  Probe (1, 2)  Probe (3) | 150 (1) – 900 (2, 3)  150 (1) – 900 (2, 3)  50 (1) – 200 (2)  250 | GTAGGATCGGAAAGCTTGGTAC  TGTTACGGCCTAAATGCTGAACT  6FAM-TGAACACCCATCCGAACAAGTAGGGTCA–TAMRA  6FAM-TGAACACCCATCCGAACAAGTAGGGTCA–BHQ1 | 84 | QT-EVE-ZM-009 |
| T25 | Forward  Reverse  Probe | 400 (1, 4) – 900 (2, 3)  400 (1, 4) – 900 (2, 3)  200 (1, 2, 4) – 250 (3) | ACAAGCGTGTCGTGCTCCAC  GACATGATACTCCTTCCACCG  6FAM-TCATTGAGTCGTTCCGCCATTGTCG–TAMRA | 102 | QT-EVE-ZM-011 |

**Table S1.5**: Oligonucleotides used as targets for the ddPCR GM cotton methods. A fluorescent marker, quencher or oligo sequence coloured in red indicates a difference with the qPCR method published in the EURL GMFF method database.

| **GM cotton event** | **Oligonucleotide^[[7]](#footnote-7)^** | **Concentration2**  **(nM)** | **Nucleotide sequence (5'->3')** | **Amplicon**  **Size (bp)** | **GMFF method** |
| --- | --- | --- | --- | --- | --- |
| LLCotton25 | Forward  Reverse  Probe (1, 3, 4)  Probe (2) | 400 (1, 2, 4) – 900 (3)  400 (1, 2, 4) – 900 (3)  200 (1, 4) – 250 (3)  200 | CAAGGAACTATTCAACTGAG  CAGATTTTTGTGGGATTGGAATTC  6FAM-CTTAACAGTACTCGGCCGTCGACCGC-TAMRA  6FAM-CTTAACAGTACTCGGCCGTCGACCGC-BHQ1 | 79 | QT-EVE-GH-002 |
| GHB614 | Forward  Reverse  Probe (1, 3)  Probe (2) | 400 (1, 2) – 900 (3)  400 (1, 2) – 900 (3)  200 (1) – 250 (3)  200 | CAAATACACTTGGAACGACTTCGT  GCAGGCATGCAAGCTTTTAAA  6FAM-CTCCATGGCGATCGCTACGTTCTAGAATT-TAMRA  6FAM-CTCCATGGCGATCGCTACGTTCTAGAATT-BHQ1 | 120 | QT-EVE-GH-006 |
| MON88701 | Forward  Reverse  Probe (1, 3)  Probe (2) | 300 (1, 2) – 900 (3)  300 (1, 2) – 900 (3)  250  250 | CATACTCATTGCTGATCCATGTAGA  AGTGTTAAACAAGTTATGTTCTAGAGC  6FAM-TTCCCGGACATGAAGCCTTAATTCAAT-TAMRA  6FAM-TTCCCGGACATGAAGCCTTAATTCAAT-BHQ1 | 84 | QT-EVE-GH-010 |
| MON1445 | Forward  Reverse  Probe (1)  Probe (2, 3) | 150 (1, 2) 900 – (3)  150 (1, 2) 900 – (3)  50  50 (2) – 250 (3) | GGAGTAAGACGATTCAGATCAAACAC  ATCGACCTGCAGCCCAAGCT  6FAM-ATCAGATTGTCGTTTCCCGCCTTCAGTTT-TAMRA  6FAM-ATCAGATTGTCGTTTCCCGCCTTCAGTTT-BHQ1 | 87 | QT-EVE-GH-003 |
| MON15985 | Forward  Reverse  Probe (1)  Probe (2, 3) | 150 (1, 2) 900 – (3)  150 (1, 2) 900 – (3)  50  50 (2) – 250 (3) | GTTACTAGATCGGGGATATCC  AAGGTTGCTAAATGGATGGGA  6FAM-CCGCTCTAGAACTAGTGGATCTGCACTGAA-TAMRA  6FAM-CCGCTCTAGAACTAGTGGATCTGCACTGAA-BHQ1 | 82 | QT-EVE-GH-005 |
| MON531 | Forward  Reverse  Probe (1)  Probe (2, 3) | 150 (1, 2) - - 900 (3)  150 (1, 2) - - 900 (3)  50  50 (2) – 250 (3) | AACCAATGCCACCCCACTGA  TCCCATTCGAGTTTCTCACGT  6FAM-TTGTCCCTCCACTTCTTCTC-TAMRA  6FAM-TTGTCCCTCCACTTCTTCTC-BHQ1 | 72 | QT-EVE-GH-004 |
| MON88913 | Forward Primer  Reverse Primer  Probe (1, 2)  Probe (3) | 500 (1, 2) – 900 (3)  500 (1, 2) – 900 (3)  100  250 | CAAATTACCCATTAAGTAGCCAAATTAC  GGCTTTGGCTACCTTAAGAGAGTC  6FAM-AACTATCAGTGTTTGACTACAT-MGBNFQ  6FAM-AACTATCAGTGTTTGACTACAT-MGBEQ | 94 | QT-EVE-GH-007 |
| T304-40 | Forward  Reverse  Probe (1, 4)  Probe (2, 3) | 400 (1, 2, 4) – 900 (3)  400 (1, 2, 4) – 900 (3)  200  200 (2) – 250 (3) | AGCGCGCAAACTAGGATAAATT  CCTAGATCTTGGGATAACTTGAAAAGA  6FAM-TCGCGCGCGGTGTCATCTATCTC-TAMRA  6FAM-TCGCGCGCGGTGTCATCTATCTC-BHQ1 | 78 | QT-EVE-GH-009 |
| GHB119 | Forward  Reverse  Probe (1, 4)  Probe (2, 3) | 400 (1, 2, 4) – 900 (3)  400 (1, 2, 4) – 900 (3)  200  200 (2) – 250 (3) | CCAGTACTAAAATCCAGATCATGCA  GAAATTGCGTGACTCAAATTCC  6FAM-CCTGCAGGTCGACGGCCGAGTAC-TAMRA  6FAM-CCTGCAGGTCGACGGCCGAGTAC-BHQ1 | 90 | QT-EVE-GH-008 |
| 3006-210-23 | Forward  Reverse  Probe (1, 4)  Probe (2)  Probe (3) | 400 (1, 2, 4) – 900 (3)  400 (1, 2, 4) – 900 (3)  150  150  250 | AAATATTAACAATGCATTGAGTATGATG  ACTCTTTCTTTTTCTCCATATTGACC  6FAM-TACTCATTGCTGATCCATGTAGATTTCCCG -TAMRA  HEX-TACTCATTGCTGATCCATGTAGATTTCCCG -BHQ1  6FAM-TACTCATTGCTGATCCATGTAGATTTCCCG -BHQ1 | 90 | QT-EVE-GH-001b |
| 281-24-236 | Forward  Reverse  Probe (1, 4)  Probe (2)  Probe (3) | 350 (1, 2, 4) – 900 (3)  450 (1, 2, 4) – 900 (3)  175  175  250 | CTCATTGCTGATCCATGTAGATTTC  GGACAATGCTGGGCTTTGTG  6FAM-TTGGGTTAATAAAGTCAGATTAGAGGGAGACAA -TAMRA  HEX-TTGGGTTAATAAAGTCAGATTAGAGGGAGACAA -BHQ1  6FAM-TTGGGTTAATAAAGTCAGATTAGAGGGAGACAA -BHQ1 | 111 | QT-EVE-GH-001a |

**Table S1.6:** Oligonucleotides used as targets for the ddPCR GM soybean methods. A fluorescent marker, quencher or oligo sequence coloured in red indicates a difference with the qPCR method published in the EURL GMFF method database.

| **GM soybean event** | **Oligonucleotide^[[8]](#footnote-8)^** | **Concentration2 (nM)** | **Nucleotide sequence (5'->3')** | **Amplicon**  **Size (bp)** | **GMFF method** |
| --- | --- | --- | --- | --- | --- |
| A2704-12 | Forward  Reverse  Probe | 400 (1, 2) – 900 (3)  400 (1, 2) – 900 (3)  200 (1, 2) – 250 (3) | GCAAAAAAGCGGTTAGCTCCT  ATTCAGGCTGCGCAACTGTT  6FAM-CGGTCCTCCGATCGCCCTTCC-TAMRA | 64 | QT-EVE-GM-004 |
| A5547-127 | Forward  Reverse  Probe (1, 3)  Probe (2) | 400 (1, 2) – 900 (3)  400 (1, 2) – 900 (3)  200 (1) – 250 (3)  200 | GCTATTTGGTGGCATTTTTCCA  CACTGCGGCCAACTTACTTCT  6FAM -CCGCAATGTCATACCGTCATCGTTGT-TAMRA  HEX-CCGCAATGTCATACCGTCATCGTTGT-BHQ1 | 75 | QT-EVE-GM-007 |
| FG72 | Forward  Reverse  Probe | 400 (1, 2) – 900 (3)  400 (1, 2) – 900 (3)  200 (1, 2) – 250 (3) | AGATTTGATCGGGCTGCAGG  GCACGTATTGATGACCGCATTA  6FAM-AATGTGGTTCATCCGTCTT-MGBNFQ | 70 | QT-EVE-GM-001 |
| DAS-68416-4 | Forward  Reverse  Probe (1)  Probe (2)  Probe (3) | 550 (1, 2) – 900 (3)  550 (1, 2) – 900 (3)  150  150  250 | GTACATTAAAAACGTCCGCAATGTG  GTTTAAGAATTAGTTCTTACAGTTTATTGTTAG  6FAM-TTAAGTTGTCTAAGCGTCAATA-MGBNFQ  VIC-TTAAGTTGTCTAAGCGTCAATA-MGBNFQ  6FAM-TTAAGTTGTCTAAGCGTCAATA-MGBEQ | 130 | QT-EVE-GM-013 |
| DAS-44406-6 | Forward  Reverse  Probe (1, 4)  Probe (2)  Probe (3) | 300 (1, 2, 4) – 900 (3)  300 (1, 2, 4) – 900 (3)  180  180  250 | TTATTGTTCTTGTTGTTTCCTCTTTAGG  CCTCAATTGCGAGCTTTCTAATTT  6FAM-ATTCGGACCTCCATGATGACCTTACCGTT-TAMRA  HEX-ATTCGGACCTCCATGATGACCTTACCGTT-BHQ1  6FAM-ATTCGGACCTCCATGATGACCTTACCGTT-BHQ1 | 99 | QT-EVE-GM-015 |
| DAS-81419-2 | Forward  Reverse  Probe (1)  Probe (2)  Probe (3) | 400 (1, 2) – 900 (3)  400 (1, 2) – 900 (3)  120  120  250 | TCTAGCTATATTTAGCACTTGATATTCAT  GCTTCAAGATCCCAACTTGCG  6FAM-ATCAACAGGCACCGATGCGCACCG-TAMRA  HEX-ATCAACAGGCACCGATGCGCACCG-BHQ1  6FAM-ATCAACAGGCACCGATGCGCACCG-BHQ1 | 105 | QT-EVE-GM-014 |
| MON89788 | Forward  Reverse  Probe (1, 2)  Probe (3) | 150 (1) – 600 (2) – 900 (3)  150 (1) – 600 (2) – 900 (3)  50 (1) – 200 (2)  250 | TCCCGCTCTAGCGCTTCAAT  TCGAGCAGGACCTGCAGAA  6FAM-CTGAAGGCGGGAAACGACAATCTG-TAMRA  6FAM-CTGAAGGCGGGAAACGACAATCTG-BHQ1 | 139 | QT-EVE-GM-006 |
| 40-3-2 | Forward  Reverse  Probe (1, 2, 4)  Probe (3) | 400 (1, 4) – 600 (2) – 900 (3)  400 (1, 4) – 600 (2) – 900 (3)  120 (1, 4) – 20 (2)  250 | TTCATTCAAAATAAGATCATACATACAGGTT  GGCATTTGTAGGAGCCACCTT  6FAM-CCTTTTCCATTTGGG-MGBNFQ  6FAM-CCTTTTCCATTTGGG-MGBEQ | 84 | QT-EVE-GM-005 |
| MON87701 | Forward  Reverse  Probe (1)  Probe (2)  Probe (3) | 400 (1, 2) – 900 (3)  400 (1, 2) – 900 (3)  250  250  250 | TGGTGATATGAAGATACATGCTTAGCAT  CGTTTCCCGCCTTCAGTTTAAA  6FAM-TCAGTGTTTGACACACACACTAAGCGTGCC-TAMRA  HEX-TCAGTGTTTGACACACACACTAAGCGTGCC-BHQ1  6FAM-TCAGTGTTTGACACACACACTAAGCGTGCC-BHQ1 | 89 | QT-EVE-GM-010 |
| 356043 | Forward  Reverse  Probe (1, 2)  Probe (3) | 750 (1, 2) – 900 (3)  750 (1, 2) – 900 (3)  200  250 | GTCGAATAGGCTAGGTTTACGAAAAA  TTTGATATTCTTGGAGTAGACGAGAGTGT  6FAM-CTCTAGAGATCCGTCAACATGGTGGAGCAC-TAMRA  6FAM-CTCTAGAGATCCGTCAACATGGTGGAGCAC-BHQ1 | 99 | QT-EVE-GM-009 |
| MON87705 | Forward  Reverse  Probe (1)  Probe (2)  Probe (3) | 450 (1, 2) – 900 (3)  450 (1, 2) – 900 (3)  250  250  250 | TTCCCGGACATGAAGCCATTTAC  ACAACGGTGCCTTGGCCCAAAG  6FAM-AAGAGACTCAGGGTGTTGTTATCACTGCGG-TAMRA  HEX-AAGAGACTCAGGGTGTTGTTATCACTGCGG-BHQ1  6FAM-AAGAGACTCAGGGTGTTGTTATCACTGCGG-BHQ1 | 86 | QT-EVE-GM-003 |
| MON87708 | Forward  Reverse  Probe (1)  Probe (2)  Probe (3) | 300 (1, 2) – 900 (3)  300 (1, 2) – 900 (3)  150  150  250 | TCATACTCATTGCTGATCCATGTAG  AGAACAAATTAACGAAAAGACAGAACG  6FAM-TCCCGGACTTTAGCTCAAAATGCATGTA-TAMRA  HEX-TCCCGGACTTTAGCTCAAAATGCATGTA-BHQ1  6 FAM-TCCCGGACTTTAGCTCAAAATGCATGTA-BHQ1 | 91 | QT-EVE-GM-012 |
| MON87769 | Forward  Reverse  Probe (1)  Probe (2)  Probe (3) | 600 (1, 2) – 900 (3)  600 (1, 2) – 900 (3)  200  200  250 | CATACTCATTGCTGATCCATGTAGATT  GCAAGTTGCTCGTGAAGTTTTG  6FAM-CCCGGACATGAAGCCATTTACAATTGAC-TAMRA  HEX-CCCGGACATGAAGCCATTTACAATTGAC-BHQ1  6FAM-CCCGGACATGAAGCCATTTACAATTGAC-BHQ1 | 87 | QT-EVE-GM-002 |
| DP 305423-1 | Forward  Reverse  Probe (1, 4)  Probe (2)  Probe (3) | 800 (1, 2, 4) – 900 (3)  500 (1, 2, 4) – 900 (3)  220  220  250 | CGTGTTCTCTTTTTGGCTAGC  GTGACCAATGAATACATAACACAAACTA  6FAM-TGACACAAATGATTTTCATACAAAAGTCGAGA-TAMRA  HEX-TGACACAAATGATTTTCATACAAAAGTCGAGA-BHQ1  6FAM-TGACACAAATGATTTTCATACAAAAGTCGAGA-BHQ1 | 93 | QT_EVE-GM-008 |
| BPS-CV127-9 | Forward  Reverse  Probe (1)  Probe (2)  Probe (3) | 400 (1, 2) – 900 (3)  400 (1, 2) – 900 (3)  100  100  250 | AACAGAAGTTTCCGTTGAGCTTTAAGAC  CATTCGTAGCTCGGATCGTGTAC  6FAM-TTTGGGGAAGCTGTCCCATGCCC-TAMRA  HEX-TTTGGGGAAGCTGTCCCATGCCC-BHQ1  6FAM-TTTGGGGAAGCTGTCCCATGCCC-BHQ1 | 88 | QT-EVE-GM-011 |

**Table S1.7:** Oligonucleotides used as targets for the ddPCR GM rapeseed, sugarbeet and rice methods. A fluorescent marker, quencher or oligo sequence coloured in red indicates a difference with the qPCR method published in the EURL GMFF method database.

| **GM rapeseed** | **Oligonucleotide^[[9]](#footnote-9)^** | **Concentration2**  **(nM)** | **Nucleotide sequence (5'->3')** | **Amplicon**  **Size (bp)** | **GMFF method** |
| --- | --- | --- | --- | --- | --- |
| MS8 | Forward Primer  Reverse Primer  Probe | 400 (1, 2) – 900 (3)  400 (1, 2) – 900 (3)  200 (1, 2) – 250 (3) | GTTAGAAAAAGTAAACAATTAATATAGCCGG  GGAGGGTGTTTTTGGTTATC  6FAM-AATATAATCGACGGATCCCCGGGAATTC-TAMRA | 130 | QT-EVE-BN-002 |
| RF3 | Forward Primer  Reverse Primer  Probe | 400 (1, 2) – 900 (3)  400 (1, 2) – 900 (3)  200 (1, 2) – 250 (3) | AGCATTTAGCATGTACCATCAGACA  CATAAAGGAAGATGGAGACTTGAG  6FAM-CGCACGCTTATCGACCATAAGCCCA-TAMRA | 139 | QT-EVE-BN-003 |
| T45 | Forward Primer  Reverse Primer  Probe | 400 (1, 2) – 900 (3)  400 (1, 2) – 900 (3)  200 (1, 2) – 250 (3) | CAATGGACACATGAATTATGC  GACTCTGTATGAACTGTTCGC  6FAM-TAGAGGACCTAACAGAACTCGCCGT-TAMRA | 123 | QT-EVE-BN-001 |
| 73496 | Forward Primer  Reverse Primer  Probe (1)  Probe (2)  Probe (3) | 600 (1, 2) – 900 (3)  600 (1, 2) – 900 (3)  200  200  250 | GTTCTTCTCTTCATAGCTCATTACAGTTTT  CAAACCTCCATAGAGTTCAACATCTTAA  6FAM-TTAGTTAGATCAGGATATTCTTG-MGBNFQ  VIC-TTAGTTAGATCAGGATATTCTTG-MGBNFQ  6FAM-TTAGTTAGATCAGGATATTCTTG-MGBEQ | 84 | QT-EVE-BN-009 |
| GT73 | Forward Primer  Reverse Primer  Probe (1, 2)  Probe (3) | 150 (1) – 900 (2, 3)  150 (1) – 900 (2, 3)  50 (1) – 200 (2)  250 | CCATATTGACCATCATACTCATTGCT  GCTTATACGAAGGCAAGAAAAGGA  6FAM-TTCCCGGACATGAAGATCATCCTCCTT-TAMRA  6FAM-TTCCCGGACATGAAGATCATCCTCCTT-BHQ1 | 168 | QT-EVE-BN-004 |
| MON 88302 | Forward Primer  Reverse Primer  Probe (1)  Probe (2)  Probe (3) | 450 (1, 2) – 900 (3)  450 (1, 2) – 900 (3)  200  200  250 | TCCTTGAACCTTATTTTATAGTGCACA  TCAGATTGTCGTTTCCCGCCTTCA  6FAM-TAGTCATCATGTTGTACCACTTCAAACACT-TAMRA  HEX-TAGTCATCATGTTGTACCACTTCAAACACT-BHQ1  6FAM-TAGTCATCATGTTGTACCACTTCAAACACT-BHQ1 | 101 | QT-EVE-BN-010 |
| **GM sugarbeet event** | **Oligonucleotide^[[10]](#footnote-10)^** | **Concentration2**  **(nM)** | **Nucleotide sequence (5'->3')** | **Amplicon**  **Size (bp)** | **GMFF method** |
| H71 | Forward Primer  Reverse Primer  Probe (1, 2)  Probe (3) | 400 (1, 2) – 900 (3)  400 (1, 2) – 900 (3)  100  250 | TGGGATCTGGGTGGCTCTAACT  AATGCTGCTAAATCCTGAG  6FAM-AAGGCGGGAAACGACAATCT-TAMRA  6FAM-AAGGCGGGAAACGACAATCT-BHQ1 | 108 | QT-EVE-BV-001 |
| **GM rice event** | **Oligonucleotide^[[11]](#footnote-11)^** | **Concentration2**  **(nM)** | **Nucleotide sequence (5'->3')** | **Amplicon**  **Size (bp)** | **GMFF method** |
| LLRICE62 | Forward Primer  Reverse Primer  Probe | 400 (1, 2, 3) – 200 (4)  400 (1, 2, 3) – 200 (4)  200 (1, 2, 4) – 250 (4) | AGCTGGCGTAATAGCGAAGAGG  TGCTAACGGGTGCATCGTCTA  6FAM-CGCACCGATTATTTATACTTTTAGTCCACCT-TAMRA | 88 | QT-EVE-OS-002 |

**Table S1.8:** Verification list for Minimum Information for Publication of Quantitative Digital PCR Experiments [The dMIQE Group, &, Huggett, J.F. (2020)]

| **Item** | **Provided/Checked** |
| --- | --- |
| **EXPERIMENTAL DESIGN** |  |
| Definition of experimental and control groups | v |
| Number within each group | v |
| Assay carried out by core lab or investigator's lab? | v |
| Power analysis | v |
| **SAMPLE** |  |
| Description | v |
| Volume or mass of sample processed | v |
| Microdissection or macrodissection | v |
| Processing procedure | v |
| If frozen - how and how quickly? | v |
| If fixed - with what, how quickly? | v |
| Sample storage conditions and duration (especially for FFPE samples) | v |
| **NUCLEIC ACID EXTRACTION** |  |
| Quantification - instrument/method | v |
| Storage conditions: temperature, concentration, duration, buffer | v |
| DNA or RNA quantification | v |
| Quality/integrity-instrument/method; e.g. RIN/RQI and trace or 3’:5’ | v |
| Template structural information | v |
| Template modification (digestion, sonication, pre-amplification etc.) | v |
| Template treatment (initial heating or chemical denaturation) | v |
| Inhibition dilution or spike; | v |
| DNA contamination assessment of RNA sample | v |
| Details of DNase treatment where performed | v |
| Manufacturer of reagents used and catalogue number | v |
| Storage of nucleic acid: temperature, concentration, duration, buffer | v |
| **REVERSE TRANSCRIPTION (If necessary)** |  |
| **dPCR TARGET INFORMATION** |  |
| Sequence accession number | v |
| Location of amplicon | v |
| Amplicon length | v |
| In silico specificity screen (BLAST, etc.) | v |
| Pseudogenes, retropseudogenes or other homologues? | v |
| Sequence alignment | v |
| Secondary structure analysis of amplicon and GC content | v |
| Location of each primer by exon or intron (if applicable) | v |
| Where appropriate, which splice variants are targeted? | v |
| **dPCR OLIGONUCLEOTIDES** |  |
| Primer sequences and/or amplicon context sequence** | v |
| RTPrimerDB Identification Number | v |
| Probe sequences** | v |
| Location and identity of any modifications | v |
| Manufacturer of oligonucleotides | v |
| Purification method | v |
| **dPCR PROTOCOL** |  |
| Complete reaction conditions | v |
| Reaction volume and amount of RNA/cDNA/DNA | v |
| Primer, (probe), Mg++ and dNTP concentrations | v |
| Polymerase identity and concentration | v |
| Buffer/kit Catalogue No and manufacturer | v |
| Exact chemical constitution of the buffer | v |
| Additives (SYBR Green I, DMSO, etc.) | v |
| Plates/tubes Catalogue No and manufacturer | v |
| Complete thermal cycling parameters | v |
| Reaction setup | v |
| Gravimetric or volumetric dilutions (manual/robotic) | v |
| Total PCR reaction volume prepared | v |
| Partition number | v |
| Individual partition volume | v |
| Total volume of the partitions measured (effective reaction size) | v |
| Partition volume variance/standard deviation | v |
| Comprehensive details and appropriate use of controls | v |
| Manufacturer of dPCR instrument | v |
| **dPCR VALIDATION** |  |
| Optimization data for the assay | v |
| Specificity (when measuring rare mutations, pathogen sequences etc.) | v |
| Limit of detection of calibration control | v |
| If multiplexing, comparison with singleplex assays | v |
| **DATA ANALYSIS** |  |
| Average copies per partition (λ or equivalent) | v |
| dPCR analysis program (source, version) | v |
| Outlier identification and disposition | v |
| Results of NTCs | v |
| Examples of positive(s) and negative experimental results as supplemental data | v |
| Where appropriate, justification of number and choice of reference genes | v |
| Where appropriate, description of normalization method | v |
| Number and concordance of biological replicates | v |
| Number and stage (RT or qPCR) of technical replicates | v |
| Repeatability (intra-assay variation) | v |
| Reproducibility (inter-assay/user/lab etc. variation) | v |
| Experimental variance or confidence interval*** | v |
| Statistical methods used for analysis | v |
| Data submission using RDML | v |
| Key: E (essential); D (desirable). | |

1. The oligonucleotide concentration or probe labelling used by a particular laboratory number is given in parenthesis [↑](#footnote-ref-1)
2. BHQ1 : Black Hole Quencher® (BHQ®) [↑](#footnote-ref-2)
3. Use by Laboratory 3 for ERM-BF412bk, ERM-BF423d and 1208-A [↑](#footnote-ref-3)
4. MGBNFQ: minor groove binder non-fluorescent quencher [↑](#footnote-ref-4)
5. MGBEQ: minor groove binder Eclipse quencher [↑](#footnote-ref-5)
6. The oligonucleotide concentration or probe labelling used by a particular laboratory number is given in parenthesis [↑](#footnote-ref-6)
7. The oligonucleotide concentrations and/or fluorophores used by a particular laboratory number is given in parenthesis [↑](#footnote-ref-7)
8. The oligonucleotide concentrations and/or fluorophores used by a particular laboratory number is given in parenthesis [↑](#footnote-ref-8)
9. The oligonucleotide concentrations and/or fluorophores used by a particular laboratory number is given in parenthesis [↑](#footnote-ref-9)
10. The oligonucleotide concentrations and/or fluorophores used by a particular laboratory number is given in parenthesis [↑](#footnote-ref-10)
11. The oligonucleotide concentrations and/or fluorophores used by a particular laboratory number is given in parenthesis [↑](#footnote-ref-11)
